# Recent Advances and Challenges in Polymer-Based Materials for Space Radiation Shielding

**DOI:** 10.3390/polym16030382

**Published:** 2024-01-30

**Authors:** Elisa Toto, Lucia Lambertini, Susanna Laurenzi, Maria Gabriella Santonicola

**Affiliations:** 1 Department of Astronautical, Electrical and Energy Engineering, Sapienza University of Rome, Via Salaria 851-881, 00138 Rome, Italy; elisa.toto@uniroma1.it (E.T.); lucia.lambertini@uniroma1.it (L.L.); susanna.laurenzi@uniroma1.it (S.L.); 2 Department of Chemical Engineering Materials Environment, Sapienza University of Rome, Via del Castro Laurenziano 7, 00161 Rome, Italy

**Keywords:** space radiation shielding, polymer-based materials, space exploration, irradiation tests, radiation transport codes

## Abstract

Space exploration requires the use of suitable materials to protect astronauts and structures from the hazardous effects of radiation, in particular, ionizing radiation, which is ubiquitous in the hostile space environment. In this scenario, polymer-based materials and composites play a crucial role in achieving effective radiation shielding while providing low-weight and tailored mechanical properties to spacecraft components. This work provides an overview of the latest developments and challenges in polymer-based materials designed for radiation-shielding applications in space. Recent advances in terms of both experimental and numerical studies are discussed. Different approaches to enhancing the radiation-shielding performance are reported, such as integrating various types of nanofillers within polymer matrices and optimizing the materials design. Furthermore, this review explores the challenges in developing multifunctional materials that are able to provide radiation protection. By summarizing the state-of-the-art research and identifying emerging trends, this review aims to contribute to the ongoing efforts to identify polymer materials and composites that are most useful to protect human health and spacecraft performance in the harsh radiation conditions that are typically found during missions in space.

## 1. Introduction

The growing interest in outer space exploration draws attention to the importance of protecting astronauts and facilities from exposure to hazardous radiation. Space radiation, comprised of high-energy particles, possesses the potential to cause ionization of atoms and molecular damage, affecting both biological tissues and spacecraft components [1,2]. In this context, the use of effective shielding materials is essential to prevent and mitigate deleterious effects on human health and on the systems that ensure a successful outcome of the mission [3].

Ionizing space radiation includes solar particle events (SPEs) and galactic cosmic radiation (GCR) [4]. GCR comprises highly energetic protons, alpha particles, electrons, and high atomic number (Z > 2) particles. In particular, the GCR spectrum consists of approximately 87% hydrogen ions (protons) and 12% helium ions (alpha particles), with the remaining 1–2% of high Z and energy (HZE) nuclei with charges from Z = 3 (lithium) to Z = 28 (nickel) [5]. Transition metals such as iron (Z = 26) are particularly difficult to shield after ionization, thus posing a risk from a biological point of view. Electrons and positrons from GCR are considered a minor biological risk during space missions since they can be shielded more easily. GCR ions pose a significant health threat to astronauts during interplanetary travels, as their energies can penetrate several centimeters of biological tissue and spacecraft materials. SPEs are produced by impulsive flares or by coronal mass ejections (CMEs). These fluxes involve electrons, protons, and other heavy-charged particles such as iron. The nature of SPEs is sporadic and unpredictable, typically associated with intense solar activity. SPEs generate energetic protons with fluences exceeding 10^9^ protons/cm^2^ [5]. Primarily composed of low linear energy transfer (LET) protons with energies up to 1 GeV/n, these can be adequately shielded by the protective structures of spacecraft. SPE dose rates vary during an event, ranging from 0 to 100 mGy/h inside a space vehicle and from zero to 500 mGy/h for astronauts during extravehicular activities (EVAs) outside of LEO. The frequency of SPEs is linked to sunspot activity, with the highest number of events occurring during periods of strong equatorial sunspot activity. Exploration missions beyond low earth orbit (LEO) that involve interplanetary travel may expose crew members to multiple SPEs, as demonstrated by the five events that occurred during the recent transit of the Mars Science Laboratory (MSL) spacecraft from Earth to Mars [6]. Moreover, the interaction of high-energy SPE protons and heavy-charged GCR particles with spacecraft structures causes onboard radiation hazards. In addition to the primary particles that can affect spacecraft, secondary particles are generated through nuclear fission reactions. This secondary radiation, including protons, alpha particles, beta particles, gamma rays, X-rays, neutrons, and heavy-charged particles, can penetrate spacecraft shielding and contribute significantly to the overall mission dose and has the potential to critically damage human tissues [5]. NASA has categorized the human health risks from space radiation into four groups: carcinogenesis, degenerative tissue risk (such as cardiovascular disease), acute and late risks to the central nervous system (CNS), and acute radiation syndromes [7]. Concerning adverse effects on spacecraft materials, they can involve the development of defects in the structure and chemical and mechanical degradation, including surface erosion and embrittlement [8]. Therefore, the engineering of suitable, high-performance radiation-shielding materials is crucial to preserve the integrity of spacecraft and the health of astronauts.

Typically, radiation shielding exploits elements with the highest charge-to-mass ratio, which are shown to be effective against HZE particles [9]. Although aluminum is commonly used in spacecraft, its exposure to GCR can lead to the production of highly penetrating secondary radiation, including neutrons and ions, which can cause electronic failures and adverse biological effects. Hydrogen, with a high charge-to-mass ratio and the absence of neutrons in its nucleus, proves effective in slowing down GCR through direct ionization [10]. Additional considerations could be taken into account. According to Bethe’s classical theory, it can be demonstrated that, for minimizing the mass of the shielding material, elements with low atomic numbers (low Z) are the most effective on a per-unit-mass basis. Conversely, if the goal is to reduce the thickness of the shielding material, elements with high atomic numbers (high Z) are most effective on a per-unit-thickness basis. A material characterized by high Z acts as a better absorber of electrons and bremsstrahlung compared to a low Z material, even if the production of bremsstrahlung is higher in materials with high atomic numbers. Nevertheless, a high Z material is less effective in proton shielding. Considering these effects, structures that incorporate both low Z and high Z materials could be promising to achieve effective radiation shielding [11].

Polymer-based materials (PBMs) have emerged as effective candidates for achieving protection against radiation while providing low-weight and tailored mechanical, thermal, and electrical properties to spacecraft components. The incorporation of suitable fillers into the polymers enables improved radiation-shielding properties. The addition of hydrogen-containing nanoparticles, as well as the incorporation of light metals, can enhance protection from GCR and SPEs [9]. Neutrons are produced as secondary particles through the interactions of GCR and SPEs with matter. Chemical elements with significant thermal neutron absorptions, such as boron, lithium, and gadolinium, can be exploited as radiation-shielding fillers [12]. Gadolinium nanoparticles, possessing the highest neutron absorption cross-section among all elements, and boron, with a substantial neutron absorption cross-section, emerge as excellent candidates for neutron shielding. Nevertheless, the use of low-Z boron compounds should be preferred to those based on gadolinium in order to avoid the generation of undesired secondary radiation, which has detrimental effects on structural materials and parasitic effects on electronic components. The neutron absorption cross-section for the isotope ^10^B is 3835 barns, and enriching boron compounds with ^10^B could enhance protection against neutrons [9]. Compounds like boron carbide (B_4_C) and hexagonal boron nitride (hBN) in nanomaterial form, particularly nano-B_4_C and nano-hBN dispersed in polymer matrix, have demonstrated enhanced thermal neutron attenuation [12]. The positive effect of using nanosized fillers can be related to their surface-to-volume ratio, which increases the interactions with radiation, enhancing the shielding effectiveness [13,14,15,16]. Boron and its low-Z compounds, like B_4_C and hBN, prove suitable for space neutron-shielding applications, while heavier elements are less convenient due to their high atomic weight, leading to fragmentation and the generation of secondary radiation.

This work provides an overview of the current developments in PBMs having radiation-shielding properties suitable for application in outer space. In the first sections, recent advances in terms of experimental and numerical investigations are discussed. In particular, the latest experimental studies on PBMs made of polyethylene (PE), polyimide (PI), polydimethylsiloxane (PDMS), and other functional matrices are described, examining the role of different fillers. The optimization of the PBM design, as well as the suitable dispersion of the reinforcement, were considered for evaluating the overall performance of the PBMs. The discussion includes the ability of PBMs to absorb incoming radiation and its effects on their properties. Numerical studies on PBMs were argued after describing the main radiation transport codes that are currently used. These codes are fundamental for evaluating the material shielding effectiveness before missions and in the selection of the most suitable radiation countermeasures. In this perspective, the strengths and weaknesses of the different codes were highlighted. Then, numerical analyses performed in GCR, SPEs, and low earth orbit (LEO) environments on different PBMs were discussed. Finally, before the concluding remarks, a section on the summary and challenges regarding the design of effective radiation-shielding PBMs was provided.

In summary, this review explores the challenges in developing multifunctional materials that are able to provide radiation protection in the harsh space environment. By identifying emerging trends in the field, this review aims to contribute to the ongoing efforts to define the PBMs that are most useful to protect humans’ health and spacecraft performance in the radiation conditions that are typically found during space exploration.

## 2. Experimental Studies

### 2.1. Polyethylene-Based Materials

Polyethylene (PE) is a versatile material successfully employed in different fields due to its ease of processing, chemical inertness, and low moisture absorption [17]. The mechanical and physical behavior of PE is strictly dependent on its crystal structure and molecular weight [18,19]. The PE properties are particularly valued for industrial storage, electronics, and in the aerospace sector due to its ability to shield against radiation [20]. PE, composed of ethylene monomers, offers effective radiation shielding due to its high hydrogen content, and this aptitude can be enhanced by embedding suitable fillers into the polymer.

PE lacks the necessary strength and thermal stability for structural use. In this regard, multifunctional fillers, such as carbon nanoparticles, have been employed to enhance the mechanical and functional properties of PE and, at the same time, improve protection from space radiation [21,22].

**Figure 1:**
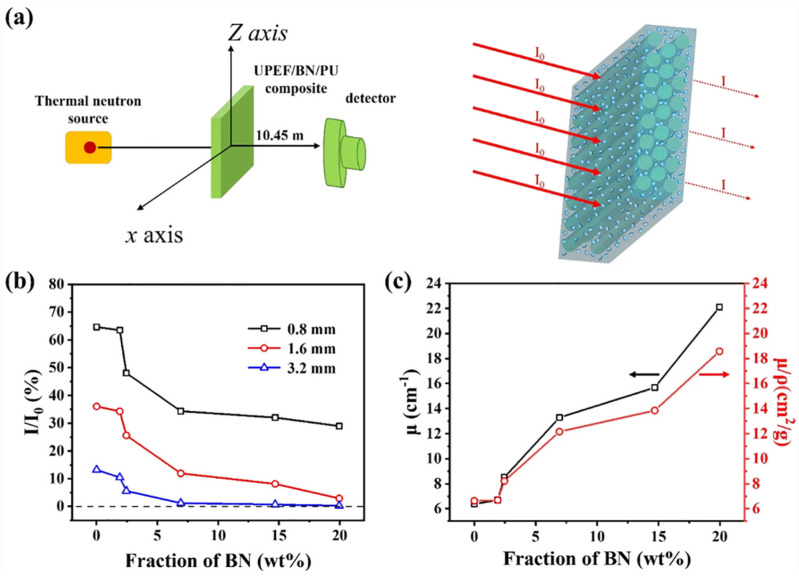
(**a**) Schematic representation of the irradiation test using a thermal neutron source; (**b**) Neutron transmission factor (I/I_0_) as a function of BN loading for UPEF/BN/PU composites with thickness of 0.8 mm, 1.6 mm, and 3.2 mm; (**c**) linear attenuation coefficient (μ), and mass attenuation coefficient (μ/ρ) as a function of BN loading for UPEF/BN/PU composites with thickness of 1.6 mm. Adapted with permission from Ref. [23].

Zhang et al. proposed the use of a composite material made of ultrahigh-molecular-weight polyethylene fiber (UPEF), boron nitride (BN), and polyurethane (PU) for effective neutron radiation shielding [23]. Neutron radiation, composed of uncharged particles, has harmful effects on human tissues, leading to diseases like cancer and cardiovascular issues [24]. Traditional neutron-shielding materials like concrete and metal have limitations, especially in aerospace applications, needing the development of new materials. In this work, UPEF, known for its mechanical properties, is combined with boron, which absorbs thermal neutrons effectively. The composite, made of UPEF, boron modified with tannic acid (TA), and PU, showed promising neutron-shielding effectiveness. In particular, the dispersion of fillers in the matrix is crucial for efficient shielding, and the modification of boron with TA improved the dispersion. The results from mechanical testing indicated high tensile strength, suggesting the potential use of the UPEF/BN/PU composite as a structural material. Figure 1a schematically represents the irradiation test. The neutron wavelength was 0.53 nm, and the sample-to-detector distance was 10.45 m. The neutron transmission factor, denoted as I/I_0_, was calculated to assess the neutron-shielding effectiveness of the UPEF/BN/PU composites at different BN loadings. I and I_0_ represent the quantities of transmitted neutron fluxes with and without the presence of the shielding composite, respectively (Figure 1a). Following the Beer–Lambert law, the attenuation of neutrons passing through the material is determined by the equation:

(1) $$\frac{I}{I_{0}}=e^{-\mu \chi }$$

where χ is the thickness of the UPEF/BN/PU composite and μ is the linear attenuation coefficient that can be expressed as follows:

(2) $$\mu =\frac{1}{\chi }\ln\frac{I_{0}}{I}$$

the mass attenuation coefficient (μ/ρ) was calculated as follows:

(3) $$\frac{\mu }{\rho }=\frac{1}{\rho \chi }\ln\left(I_{0}/I\right)$$

where ρ is the density of the sample. The results unveiled that the neutron radiation intensity decreases by approximately 25%, 63%, and 85% when passing through UPEF/BN/PU-0 with thicknesses of 0.8, 1.6, and 3.2 mm, respectively (Figure 1b). Considering samples without BN, the neutron attenuation results from the high hydrogen content in UPEF. The findings align with theoretical predictions that emphasize the effective neutron-shielding properties of materials rich in hydrogen. For the UPEF/BN/PU samples loaded with BN at 7 wt%, the neutron-shielding capability improves, as indicated by the decreasing of the I/I_0_ value. This improvement is attributed to the ability of boron to absorb neutrons through nuclear reactions with the boron nucleus in BN. Neutrons are sequentially moderated by hydrogen and absorbed by boron. However, above 7 wt% of BN loadings, the efficiency of neutron shielding decreases. This suggests that continuously adding BN fillers may not enhance neutron-shielding efficiency and could potentially affect the mechanical properties of the composites. Moreover, the thickness of the UPEF/BN/PU composite significantly influences neutron-shielding performance. For BN content below 7 wt%, I/I_0_ for UPEF/BN/PU with a thickness of 1.6 mm is approximately 25–30% lower than that of UPEF/BN/PU with a thickness of 0.8 mm. With increasing BN content, the difference in the I/I_0_ value between UPEF/BN/PU composites with different thicknesses decreases. Figure 1c indicates that both μ and μ/ρ increase as the BN content rises. Definitively, an optimal BN content of 7 wt% is identified, balancing neutron-shielding efficiency and mechanical properties. Hence, the UPEF/BN/PU composite can be considered a suitable candidate for radiation shielding in aerospace structures.

Neutron exposure experiments were conducted by Herrman et al. on high-density polyethylene (HDPE)-based composites [25]. Boron carbide (BC) and boron nitride (BN) with particle sizes less than 10 μm were chosen as fillers and added to injection-molding-grade HDPE. Irradiation tests were performed using a 1-Curie Americium-Beryllium neutron source. A dose rate of 0.6 mSv/h/GBq at a distance of 1 m was applied for 15 min, with the source positioned 50 cm away from the detector, and counts were recorded at 10 s intervals. The neutron exposure tests were conducted using bare indium foil and bare indium foil/sample pairings. Bare indium foil was used as a reference due to its high neutron absorption cross-section. The indium foil/sample pairings included bare indium foils with 1%, 5%, and 30% BN samples. The neutron exposure tests aimed to determine the mass absorption cross-section (μ/ρ) for thermal neutrons and the effectiveness of shielding. The results suggested that higher BN content leads to lower initial radiation detected, and mass absorption (shield effectiveness) increased with lower initial activity. Atomic force microscopy (AFM) studies revealed that HDPE-BN blends were reasonably uniform at low BN concentrations, while higher percentages reduced uniformity, potentially due to the lubricating effect of BN. Compressive strength was observed to decrease initially with BN addition, but higher amounts of BN induced an increase in the strength. The presence of boron nitride in the sample was found to influence mass absorption, confirming its role in shaping the shielding properties of the material. Based on these findings, the HDPE/BN composites can be considered for potential use in aerospace due to their advantageous mechanical and radiation-shielding properties.

Zaccardi et al. fabricated multifunctional nanocomposites using medium-density polyethylene (MDPE) loaded with multiwalled carbon nanotubes (MWCNTs), graphene nanoplatelets (GNPs), and hybrid MWCNT/GNP fillers [22]. Electrical properties, chemical structure, thermal behavior, wettability, and morphology were investigated before and after proton irradiation. In particular, the samples were irradiated for 294 s, with an energy of 64 MeV and a current of 1 nA, resulting in a total dose of 50 Gy. According to the U.S. Center for Disease Control and Prevention’s information on acute radiation syndrome, a dose of 50 Gy is known to induce the fatal collapse of the human cardiovascular and central nervous systems. This dosage exceeds the acceptable exposure limits for astronauts [26]. The experiments were conducted at the Crocker Nuclear Laboratory of the University of California (Davis, CA, USA). FTIR analyses revealed a decrease in crystallinity (Xc) after irradiation, more pronounced in the GNP-filled nanocomposites. Thermal analysis using differential scanning calorimetry (DSC) confirmed the decrease in crystallinity and unveiled thermal stability after irradiation. Contact angle measurements indicated a decrease in hydrophobicity after proton exposure, whereas morphological analysis by SEM highlighted surface erosions after irradiation. The results indicated that the MDPE/MWCNT 5 wt% nanocomposite maintains thermal stability, a hydrophobic behavior, and negligible changes in crystallinity, making it a promising shielding material in high-ionizing-radiation environments, such as space.

**Figure 2:**
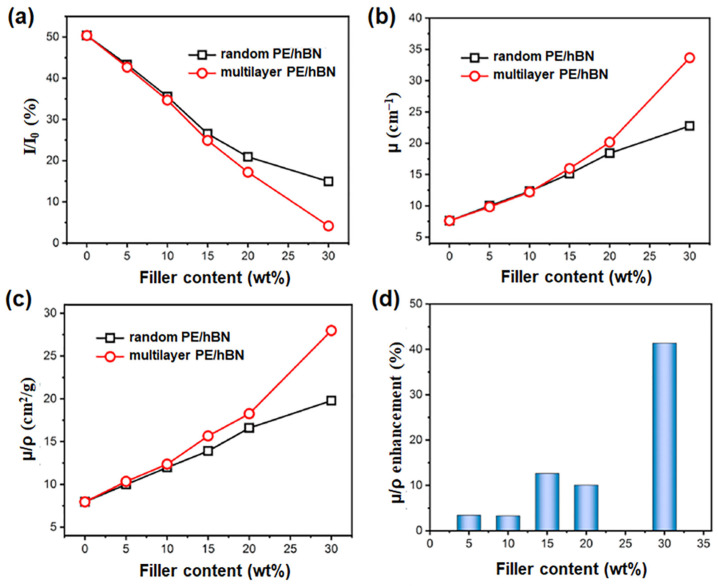
(**a**) Neutron transmission factor (I/I0), (**b**) linear attenuation coefficient (μ), and (**c**) mass attenuation coefficient (μ/ρ) as a function of hBN content for PE/hBN films. (**d**) μ/ρ enhancement of multilayer and random PE/hBN films. Adapted with permission from Ref. [27].

A multilayer composite material was developed by alternately stacking layers of high-density polyethylene/hexagonal boron nitride (HDPE/hBN) and low-density polyethylene (LDPE) [27]. The neutron-shielding ability of these PE/hBN composites was evaluated after irradiation experiments using neutrons with a wavelength (*λ*) of 0.53 nm and a spread Δλ/*λ* = 16%. The composites were fabricated by a two-step hot-pressing process, where HDPE/hBN and LDPE layers were individually hot-pressed into slices and then stacked alternately and hot-pressed to form multilayer composite films. This strategy aimed to align hBN along the in-plane direction for improved performance. The neutron-shielding effectiveness was evaluated using neutron transmission factor (I/I_0_), linear attenuation coefficient (μ), and mass attenuation coefficient (μ/ρ). The results indicate that the neutron radiation intensity decreases by ~50% when passing through PE films (Figure 2a), and this can be justified by the high hydrogen content in PE. The incorporation of hBN into the PE matrix enhances the neutron-shielding performance, as proven by a decrease in the I/I_0_ value at increasing hBN loadings. This result can be ascribed to the synergistic attenuation effect of hydrogen and boron atoms. The optimal I/I_0_ value is achieved at 4.16% for the multilayer composite with a 30 wt% hBN content, indicating that ~95.84% of neutron radiation is shielded when passing through the composite. Comparatively, at filler loadings below 15 wt%, multilayer composites exhibit similar shielding ability to random composites, possibly due to incomplete coverage of hBN. Considering filler loadings above 20 wt%, the multilayer composites showed superior shielding ability compared to random composites. At 30 wt% filler content, the I/I_0_ for multilayer PE/hBN decreases to 4.16%, much lower than the random PE/hBN value of 14.95%. Figure 2b,c reveal that both μ (linear attenuation coefficient) and μ/ρ (mass attenuation coefficient) increase with higher hBN content, and the multilayer PE/hBN values exceed those of random PE/hBN at the same hBN content. Figure 2d shows the μ/ρ enhancement calculated by Δμ/ρ = (μ/ρ_m_ − μ/ρ_r_)/(μ/ρ_r_), where μ/ρ_m_ and μ/ρ_r_ are referred to multilayer PE/hBN and random PE/hBN, respectively. At low filler content, Δμ/ρ is small, indicating that the multilayer structure has a minimal impact on neutron-shielding enhancement. However, with a 30 wt% hBN content in the multilayer composite, Δμ/ρ reaches 41.37%, demonstrating the effective improvement of neutron-shielding performance with a high filler content. A critical hBN content (30 wt%) was detected for the noticeable enhancement of the multilayer structure compared to the random composite. Overall, the performance of the PE/hBN multilayer sample suggests its potential application in neutron shielding and thermal management in fields such as aerospace.

Composite materials made of high-density polyethylene (HDPE) filled with Al_2_O_3_, Fe_2_O_3_, and PbO were fabricated and tested under γ-radiation [28]. A 60 KBq source of ^226^Ra was used to obtain the γ-rays beam at different energies: 0.295, 0.352, 0.609, 1.12, and 1.747 MeV. The following parameters were used to compare the radiation-shielding effectiveness of the composites: linear attenuation coefficient (μ), transmission factor (TF), mean free path (MFP), half-value layer (HVL), and radiation protection efficiency (RPE). These parameters are expressed as follows:

(4) $$TF=\frac{I}{I_{0}}$$

(5) $$MFP=\frac{I}{\mu }$$

(6) $$HVL=\frac{\ln2}{\mu }$$

(7) $$RPE=\left(1-e^{-\mu t}\right)\times 100$$

The following materials were prepared and tested: pure HDPE, HDPE + 30% Al_2_O_3_, HDPE + 30% Fe_2_O_3_, HDPE + 10% PbO, HDPE + 30% PbO, and HDPE + 50% PbO. The values of μ decrease at increasing radiation energies. A noticeable decrease in μ was observed for radiation energies below 0.609 MeV, and this can be ascribed to the dominance of the photoelectric effects in the material. Above 0.609 MeV, the dominance of Compton scattering takes place. The results showed that the samples containing PbO have superior attenuation capacity and efficiency compared to those loaded with Al_2_O_3_ and Fe_2_O_3_. Among the composites, the one loaded with 50% PbO achieved the best results, demonstrating the highest value of μ and the smallest values of MFP, HVL, and TF for all the tested radiation energies. Hence, these composites can be considered effective radiation-shielding materials to be potentially applied in fields requiring high γ-ray attenuation, such as space.

### 2.2. Polyimide-Based Materials

Polyimides (Pis) are a class of high-performing polymers showing outstanding thermal stability, chemical and radiation resistance, and suitable mechanical and dielectric properties [29,30,31,32]. Pis can be considered neutron moderators since their structure includes carbon, hydrogen, nitrogen, and oxygen, which mitigate the generation of secondary particles after collision with neutrons. The radiation-shielding effectiveness of Pis has been successfully enhanced by the incorporation of nanomaterials, such as bismuth oxide and boron nitride.

Pavlenko et al. fabricated polyimide-based composites filled with bismuth oxide (Bi_2_O_3_) and tested their radiation-shielding behavior under γ-ray exposure [33]. The Bi_2_O_3_ particles were modified with polymethylphenylsiloxane (PMPS) to achieve a uniform distribution in the composites. The irradiation tests were performed at 400 keV and 662 keV, using radionuclides ^192^Ir (400 keV) and ^137^Cs (662 keV) as sources. The results demonstrated that the incorporation of Bi_2_O_3_ significantly improved the thermal stability of the composites. Moreover, the modified Bi_2_O_3_ particles showed hydrophobic behavior, enhancing their distribution in the non-polar PI matrix. Composites produced by hot-pressing exhibited higher density and microhardness compared to those produced by cold-pressing, indicating a more uniform distribution of fillers. The radiation-shielding properties of the composites were assessed experimentally and theoretically, unveiling a high-radiation-protective behavior. In particular, the results showed that the mass attenuation coefficient (μ_m_) increases linearly with the Bi_2_O_3_ loading (0–60 wt%). Overall, the incorporation of PMPS-modified Bi_2_O_3_ into the PI matrix and the hot-pressing method proved effective for fabricating attractive materials for space technology and radiation protection.

Baykara et al. developed composite materials with shielding properties against both neutrons and γ-rays. A thermoplastic polyimide was used as a matrix and filled with gadolinium oxide (Gd_2_O_3_) and hexagonal boron nitride (hBN) nanoparticles [34]. The neutron-shielding properties of hBN/Gd_2_O_3_/PI samples with different filler loadings were assessed after irradiation with a ^239^Pu-Be neutron source. Neutron transmission factors were determined experimentally by measuring the ratio of incident (I_0_) and transmitted (I) neutron fluxes. The macroscopic cross-section (Σ) and mass attenuation coefficient (μ/ρ) were computed based on the count rate (cps) for neutrons and the gamma dose rate (μR/h) for gamma radiation measured during the experiments. The source used for experiments generates both neutron and gamma radiation, and suitable measurements were conducted to distinguish and evaluate the shielding efficiencies of the nanocomposites against each type of radiation. The results unveiled that samples exhibited an exponential decrease in neutron flux attenuation, with fluctuations observed as the thickness increased. The fluctuations were attributed to the interaction of neutrons with nanoparticles within the shielding materials. The nanocomposite with 11 wt% hBN showed the highest neutron-shielding performance. Considering samples at high loadings of Gd_2_O_3_, the neutron transmissions exhibited fluctuations dependent on the thickness of the shield material. To validate the results, neutron permeability experiments for the nanocomposite with 11 wt% hBN/3 wt% Gd_2_O_3_/PI were repeated at different thicknesses. In this case, results demonstrated consistency in neutron-shielding efficiency between the initial and repeated experiments. The gamma shielding ability of the nanocomposites was also thoroughly investigated. The experiments involved measuring gamma dose rates resulting from the interaction between the neutron source and the neutron-shielding material. The nanocomposites showed an exponentially decreasing behavior in gamma transmission, with transmission percentages superior to that of neat polyimide. Further analysis involved the calculation of macroscopic cross-section and mass attenuation coefficient values for both neutron and gamma rays. The macroscopic cross-section values for nanocomposites ranged between 0.1898 and 0.4052 cm^−1^, exhibiting a significant improvement compared to neat polyimide (0.1316 cm^−1^). In summary, the findings of this experimental study demonstrated the multifunctional properties of the nanocomposites, with high efficacy in attenuating both neutron and gamma radiation due to the interplay between hBN and Gd_2_O_3_. The results indicate that these nanocomposites are promising materials for applications requiring effective protection against radiation sources in fields such as aerospace.

**Figure 3:**
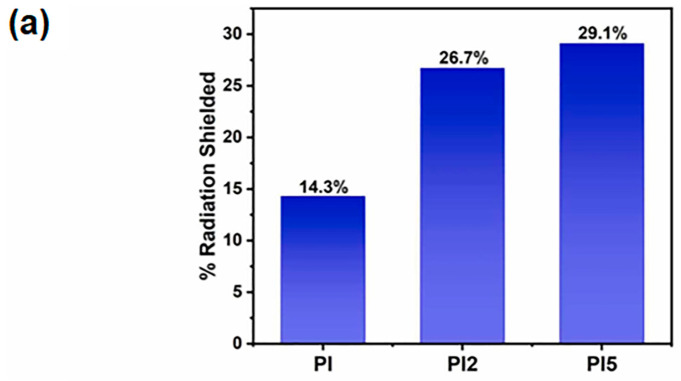

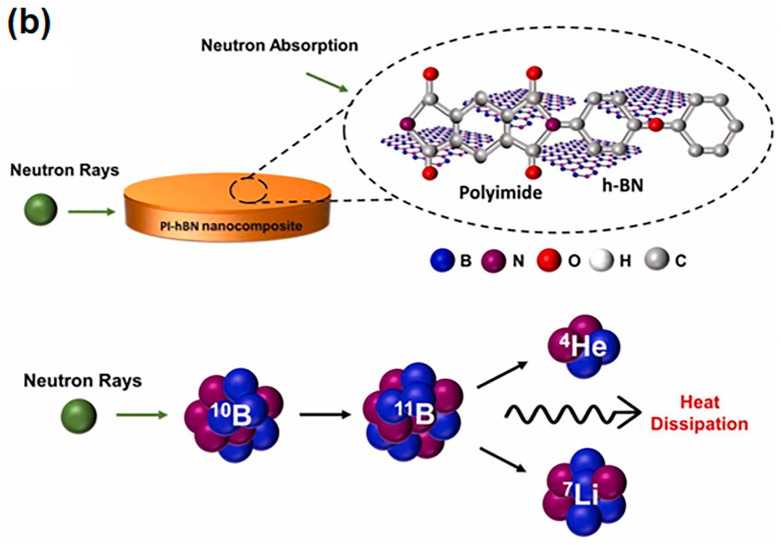
(**a**) Percentage of radiation shielded by neat PI, 2 wt% hBN/PI, and 5 wt% hBN/PI nanocomposites; (**b**) schematic representation of the neutron-shielding mechanism of hBN/PI nanocomposites. Adapted with permission from Ref. [35].

Polyimide-hexagonal boron nitride (PI-hBN) nanocomposites were fabricated using direct forming technology (DF) to enhance their tribological and radiation-shielding properties [35]. The process involved the incorporation of hexagonal boron nitride (hBN) nanoparticles at concentrations of 2 wt% and 5 wt% into a polyimide matrix. The ball milling technique was employed to minimize nanoparticle agglomeration and ensure the uniform distribution of hBN within the PI matrix. The neutron-shielding effectiveness of the samples was examined using an americium (^241^Am)-beryllium (^9^Be) neutron source. The assessment of neutron shielding involved the examination of the linear absorption coefficient (μ) and mass absorption coefficient (μ/ρ). In particular, μ was used to quantify the extent of incident radiation attenuation, normalized by thickness, whereas μ/ρ expresses the attenuated radiation normalized by both density and thickness, as indicated by Equation (3). Quantitative measurements of neutron transmission through the nanocomposites were performed, and the results highlighted a significant decrease in neutron flux as a function of the hBN content. The 2 wt% and 5 wt% hBN-loaded PI nanocomposites exhibited significantly lower neutron transmission compared to the neat PI matrix. In particular, the linear and mass absorption coefficients exhibited substantial improvement, increasing by 1.9 and 2.2 times for 2 wt% and 5 wt% hBN-loaded PI, respectively, in comparison to the neat PI. Figure 3a shows that the percentage of radiation shielded by 2 wt% and 5 wt% hBN-loaded PI increased by 26.7% and 29.1%, respectively, in comparison to the unloaded PI. These results confirm the efficacy of hBN as a neutron-absorbing component within the composite. As schematically represented in Figure 3b, the presence of hydrogen-rich atoms in PI facilitates the deceleration of neutron beams during collisions and contributes to neutron absorption within the matrix. The shielding effectiveness of the PI matrix is further heightened by hBN, where boron atoms play a role in thermal neutron capture. Neutron absorption occurs when low-energy thermal neutrons irradiate the stable ^10^B isotope in hBN. As a result, reactions such as nuclear capture and fission take place, leading to the formation of alpha particles (^4^He) and (^7^Li) nuclei, accompanied by the generation of heat. Subsequently, the generated heat is dissipated through hBN within the PI matrix. Overall, the nanocomposites demonstrated superior neutron-shielding properties, unveiling their potential suitability for applications in space missions where exposure to neutron radiation is a critical concern.

Cherkashina et al. focused on the impact of electron irradiation on the structural and property changes in polyimide materials [36]. Composite samples based on PI track membranes and nanodispersed lead (70 wt%) were fabricated. The impact of fast electrons with energies ranging from 1 to 5 MeV was evaluated on the neat PI and on the PI composite. The irradiation employed a one-sided mode, preceded by thermal treatment of the samples in a vacuum oven at 180 °C for 3 h. To assess the absorbed dose distribution and effective electron range, tests were conducted by incrementally increasing sample thickness. Samples of 25 μm thickness were tightly stacked, and a detector was placed behind them. The effective electron range was determined by the total film thickness at which the detector ceased detecting radiation. All samples received a total dose of 10 MGy. The effective range of electrons detected in PI and PI composite is directly proportional to initial electron energy and increases with energy. Enhanced radiation-shielding characteristics of the composite, compared to PI, are attributed to the structure of composites. Contact between the track membrane and metallic lead results in a contact potential difference, creating an electric field preventing electron transition. The near-contact layer of the composite enriches with electrons, increasing conductivity. For high-energy electrons, the increased electron density in the near-contact layer leads to a higher probability of scattering at larger angles, causing ionization and radiation losses. This results in a significant reduction in electron flux in the PI composite. Overall, results showed that the penetration depth of electrons into PI is greater than in the composite. The tensile strength of both materials decreases slightly after irradiation, and the electrical properties of the composite remain largely unaffected. The findings of this experimental study suggested the potential use of the PI/lead composite in space, offering protection against cosmic radiation.

**Figure 4:**
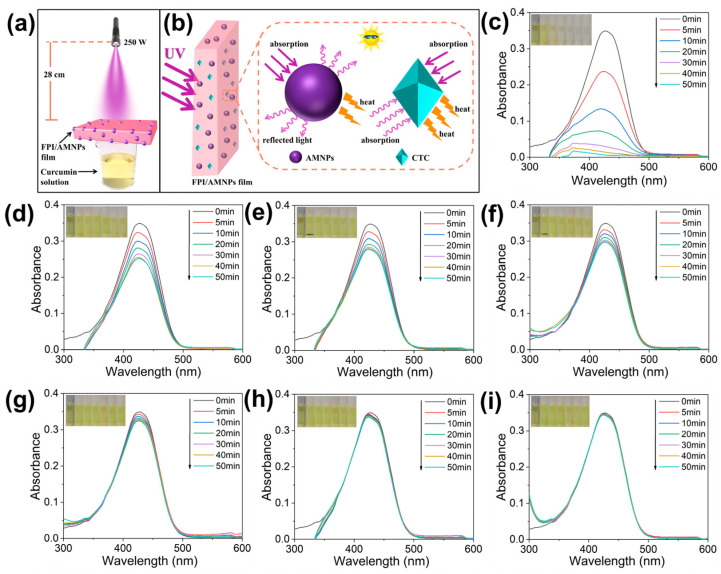
Schematic representation of (**a)** experiment and of (**b**) UV interaction with the FPI/ANMP composites. (**c**) UV-vis spectra of curcumin without shielding. UV-vis spectra of curcumin covered by (**d**) FPI, (**e**) FPI/AMNPs-0.1%, (**f**) FPI/AMNPs-0.3%, (**g**) FPI/AMNPs-0.5%, (**h**) FPI/AMNPs-0.7%, and (**i**) FPI/AMNPs-1%. The inserted images show the corresponding shade of curcumin solution from 0 to 50 min. Adapted with permission from Ref. [37].

Ultraviolet (UV)-shielding materials based on a highly fluorinated polyimide (FPI) filled with allomelanin nanoparticles (AMNPs) were developed by Li et al. [37]. The presence of fluorinated groups in FPI increases porosity and decreases density, expanding the propagation path of UV and enhancing UV-shielding performance. The results demonstrate improved mechanical and UV-shielding properties with the synergistic absorption of UV by FPI and AMNPs. In this study, curcumin was used to evaluate the UV-shielding performance of the composites through UV-vis measurements, as schematically represented in Figure 4a. Figure 4b schematically represents the UV interaction with the FPI/ANMPs composite. Curcumin was chosen due to its high instability under UV irradiation, leading to the decomposition of the α-carbon in its structure into aldehydes, further oxidizing into acids [38]. In the blank control group (Figure 4c), the absorbance of curcumin dropped to zero after 50 min of irradiation. This correlated with a color change from dark yellow to colorless, confirming complete degradation. In contrast, curcumin covered by a pure FPI film only experienced partial degradation, with a residual rate reaching 72.2% (Figure 4d), accompanied by a slight solution fade. When curcumin was shielded by FPI + 0.1% AMNPs, FPI + 0.3% AMNPs, FPI + 0.5% AMNPs, FPI + 0.7% AMNPs, and FPI + 1% AMNPs films, the concentration of the curcumin solution decreased less with an increase in AMNPs content (Figure 4e–i). Simultaneously, the color change in the curcumin solution was not gradually evident, consistent with the initial solution.

**Figure 5:**
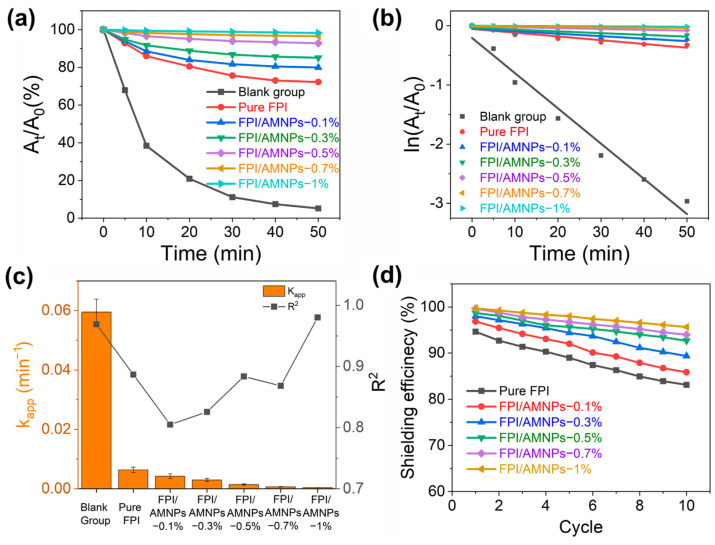
(**a**) Decay curves of the absorption intensity of the curcumin solution at 425 nm with and without shielding by FPI/AMNPs composites; (**b**) dynamic reaction rate curve of curcumin decomposition; (**c**) kinetic reaction rate constant and linear correlation coefficient curves; (**d**) shielding efficiency after 0–10 cycles of UV exposure. Adapted with permission from Ref. [37].

To quantify the UV-shielding efficiency of FPI/AMNP films, decay and dynamic reaction rate curves for curcumin decomposition were plotted (Figure 5a,b). Figure 5a shows that curcumin decays most rapidly in the blank group, while the decay rate was significantly reduced when shielded by pure FPI, indicating effective UV shielding. Moreover, the attenuation degree of curcumin decreased with increasing AMNP content, demonstrating enhanced UV shielding favored by AMNPs. The relationship between ln (A_t_/A_0_) and time (t) confirmed first-order linear reaction kinetics for curcumin decomposition (Figure 5b). The largest k_app_ belonged to the blank group, and others corresponded to curcumin covered by pure FPI, FPI + 0.1% AMNPs, FPI + 0.3% AMNPs, FPI + 0.5% AMNPs, FPI + 0.7% AMNPs, and FPI + 1% AMNPs, respectively. The results indicated that the UV-shielding effect of FPI/AMNP films was proportional to the accumulation of AMNPs, highlighting the synergistic effect of FPI and AMNPs in enhancing UV shielding. A considerable amount of charge transfer complexes (CTCs) between molecular chains of FPI and excellent UV absorption by AMNPs contribute to the enhanced UV-shielding properties of the composite films. The R^2^ value, consistently above 0.87, indicates a well-fitted linear relationship for this reaction (Figure 5c). Material reusability is crucial in practical use, with UV-shielding efficiency increasing with higher AMNP content (Figure 5d). After 10 recycling tests, FPI/AMNPs-1% exhibited a 13% amplification in UV-shielding efficiency compared to pure FPI. Furthermore, pure FPI showed a 12% reduction in UV-shielding efficiency, while FPI/AMNPs-1% showed only a 4% reduction. These results highlight the effective prolongation of UV-shielding life with the addition of AMNPs. In summary, FPI/AMNP films demonstrated excellent UV-shielding properties and reusability, indicating them as potential candidates for ultraviolet shielding in space.

### 2.3. Polydimethylsiloxane-Based Materials

PDMS shows advantageous properties such as flexibility, suitable thermal stability, chemical inertness, and low cost [39,40,41,42]. These properties have been extensively exploited for the development of membranes, enclosures, microfluidic structures, and sensors [43,44,45,46,47]. Low-Z fillers have been successfully embedded in PDMS-based matrices, obtaining promising radiation-shielding materials that can be potentially exploited in the space environment.

PDMS nanocomposites filled with tungsten oxide (WO_3_) and barium oxide (BaO) were exposed to ^137^Cs, ^241^Am, and ^60^Co with energies ranging from 0.059 to 1.333 MeV [48]. The following samples were fabricated: 100 wt% PDMS (S-1), 60 wt% PDMS + 40 wt% WO_3_ (S-2), 60 wt% PDMS + 40 wt% BaO (S-3), double layers of S-2 and S-3 (S-4), and double layers of S-3 and S-2 (S-5). The results showed that at the lowest energy level (0.059 MeV), the sample S-3 exhibited the highest µ (5.66 cm^−1^), followed by S-4 (4.07 cm^−1^), while S-1 (neat PDMS) had the smallest µ (0.29 cm^−1^). The addition of WO_3_ and BaO consistently improved the µ values, indicating enhanced radiation attenuation for the composites. The transmission factor (TF) values obtained for the samples confirmed that the addition of WO_3_ and BaO contributed to increased radiation attenuation, particularly at lower energies. However, as energy increases, the differences in TF between samples decrease. The double-layered samples (S-5 and S-6) showed better TF, especially at low energies, compared to single-layered samples. The half-value layer (HVL), representing the material thickness required to reduce radiation intensity by 50%, was calculated. The results showed that S-1 (neat PDMS) had the highest HVL, confirming poorer shielding performance compared to samples containing WO_3_ and BaO. Sample S-2, loaded with 40 wt% WO_3_, demonstrated better radiation attenuation efficiency. Samples containing higher percentages of WO_3_ and BaO showed lower MFP values, indicating improved shielding efficiency. In summary, these flexible composites exhibited suitable performance against γ-ray radiation that could be potentially exploited in different fields, such as aerospace.

**Figure 6:**
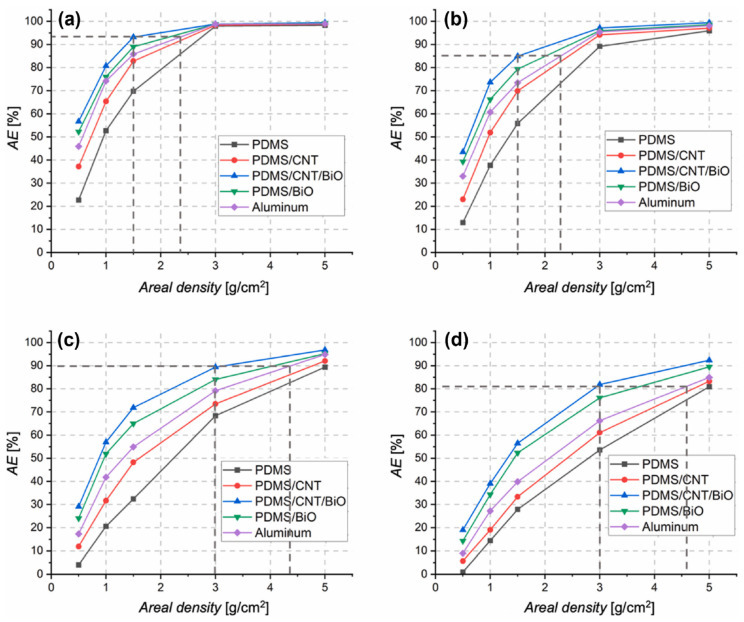
Percentage of electron attenuation efficiency (AE%) of PDMS, PDMS/CNT, PDMS/CNT/BiO, and aluminum samples tested under electron beam energies of (**a**) 9, (**b**) 12, (**c**) 16, and (**d**) 20 MeV in attenuation mode. Dashed lines: AE% difference between samples with the same areal density (horizontal lines) and areal densities difference for the same AE% (vertical lines). Adapted with permission from Ref. [49].

Cheraghi et al. fabricated PDMS-matrix nanocomposites filled with bismuth oxide (Bi_2_O_3_) and multiwalled carbon nanotubes (MWCNTs) and tested their shielding properties against high-energy electron beam for potential application in space [49]. Samples of pure PDMS and nanocomposites filled with 30 wt% of Bi_2_O_3_ and 3 wt% of MWCNTs were prepared and tested under electron beam energies of 9, 12, 16, and 20 MeV in attenuation mode. These values were selected according to the electron beam energies reported for outer space. The shielding behavior of the samples was compared with that of aluminum, which was considered as the reference material. During the irradiation tests, each sample received a 100 cGy dose at a rate of 1000 cGy/min. The percentage of electron attenuation efficiency (AE%), equivalent to the RPE factor mentioned in Section 2.1, was calculated as follows:

(8) $$AE\left(\%\right)=\frac{C_{0}-C_{t}}{C_{0}}\times 100$$

where C_0_ and C are the intensities of the original and transmitted electrons measured using an ionizing chamber. The results unveiled that PDMS/BiO and PDMS/CNT/BiO nanocomposites have better shielding properties than aluminum, pure PDMS, and PDMS/CNT samples for all areal densities (Figure 6).

For all the samples, the electron attenuation efficiency showed a similar trend for all energies, with AE% values that increase at increasing areal densities. PDMS/CNT/BiO samples showed the highest weight advantages and AE% values at any electron beam energies. The difference between the attenuation values of PDMS/CNT/BiO and aluminum decreases for higher areal densities. Considering the density values of 0.5, 1, and 1.5 g/cm^2^, the PDMS/CNT/BiO sample showed better shielding capabilities than aluminum. Therefore, these PDMS-based materials can be potentially applied in space as shielding materials instead of aluminum, which has disadvantages such as heavy weight and generation of secondary electrons.

Borjanovic et al. fabricated PDMS-matrix nanocomposites filled with single-walled carbon nanotubes (SWCNTs), detonation nanodiamond (DND), and zinc oxide (ZnO) and tested their radiation-shielding behavior under proton exposure [50]. Irradiation experiments were performed using a 2 MeV proton beam and low fluence conditions with currents ranging from 30 to 100 nA. The samples were irradiated in the fluence range of 10^13^ to 10^15^ protons/cm^2^ in four regions (D1-D4) with different conditions. In particular, the fluence associated with regions 1 (D1) and 2 (D2) is 5.7 × 10^13^ protons/cm^2^. In region 1, a beam current of 30 nA was used, while in region 2, a beam current of 50 nA was applied. The total charge for both regions 1 and 2 was kept constant at 5.6 μC. For region 3 (D3), a fluence of 5.7 × 10^14^ protons/cm^2^ was achieved with an 80 nA beam current, resulting in a total charge of 56 μC. In region 4 (D4), a fluence of 1 × 10^15^ protons/cm^2^ was provided using a 100 nA beam current, with a total charge of 100 μC. The proton irradiation was conducted at room temperature. The study emphasized the influence of particle size on ionizing radiation protection, with smaller particles showing enhanced stability in high irradiation environments. Raman and FTIR-ATR spectra revealed that PDMS-DND nanocomposites with 40 nm DND aggregates were more stable under high proton fluences compared to pure PDMS or other nanocomposites. The introduction of ZnO nanoparticles into the PDMS matrix (PDMS-ZnO) showed even better high-energy ionizing radiation resistance than the DND-filled materials. The multifunctionality of ZnO, including energy absorption and dissipation, contributed to its effectiveness in protecting the nanocomposite under high fluences. Moreover, results indicated that PDMS-SWCNT nanocomposites provided protection similar to other nanofillers under low proton fluences, while their effectiveness decreased at higher fluences (D3, D4). In conclusion, the nanocomposites with smaller filler sizes and those with ZnO exhibited the best shielding performance against high-energy proton irradiation, making them suitable candidates for applications in high-radiation environments.

**Figure 7:**
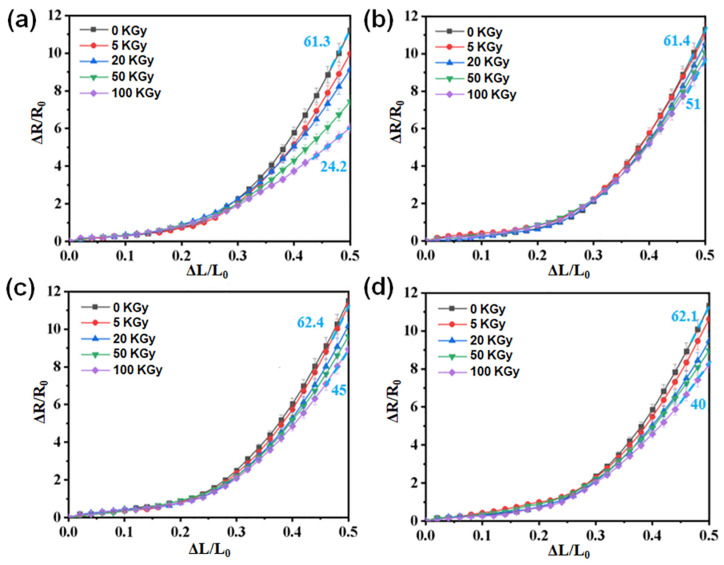
Plots from force-sensitive tests after different doses of gamma irradiation performed on (**a**) CNT sponge/PDMS samples and CNT sponge/PDMS samples containing tungsten oxide with particle sizes of (**b**) 50 nm, (**c**) 100 nm, and (**d**) 1 μm. Adapted with permission from Ref. [51].

Han et al. proposed a novel force-sensitive structure resistant to γ-rays exposure, employing a sandwich configuration made of tungsten oxide (WO_3_), PDMS, and carbon nanotube (CNT) sponge [51]. Tungsten oxide was used as a gamma-ray shielding material, while the CNT sponge establishes a conductive network, and PDMS acts as a flexible substrate. Irradiation experiments were conducted on four samples using ^60^Co-γ rays with an energy value of 1.25 MeV. The force sensitivity of samples irradiated with different doses (0, 5, 20, 50, 100 KGy) was assessed and shown in Figure 7. Sensitivity was defined as (ΔR/R_0_)/ε, where ε was indicated as ΔL/L_0_. As reported in Figure 7a, the sensitivity of the CNT sponge/PDMS sample decreased from 61.3 to 24.2, indicating a reduction of approximately 60% after gamma irradiation. Conversely, samples containing tungsten oxide particles exhibited a lower sensitivity attenuation after exposure (Figure 7b–d). The sensitivity of the samples containing WO_3_ with particle sizes of 50 nm (Figure 7b), 100 nm (Figure 7c), and 1 μm (Figure 7d) decreased by 17%, 28%, and 35.6%, respectively. With an increase in the WO_3_ particle size, the fluctuations in the force-sensitive curves after irradiation gradually became more pronounced. A more significant impact of gamma rays on the force-sensitive performance of the samples was observed when larger WO_3_ particles were used. Differently, the use of smaller particles of WO_3_ induced more protection against gamma rays, and this can be ascribed to the presence of more gaps in the composite. Using smaller WO_3_ particles, the specific surface area increases, leading to the formation of more gamma loss interfaces and reducing the impact of gamma rays on the composite. Overall, this work proposed a promising flexible stress sensor with gamma-ray-shielding effectiveness, which could be potentially used in space.

### 2.4. Other PBMs

Prabhu et al. developed epoxy-based materials filled with micro and nano-tantalum oxide (Ta_2_O_5_) particles and demonstrated their effectiveness in γ-ray shielding [16]. A diglycidyl ether of bisphenol A (DGEBA) epoxy resin was used as a matrix. The study determined μ/ρ values for composites loaded with 10%, 20%, and 30 wt% of filler and irradiated using energy values of 0.356, 0.511, 0.662, 1.173, 1.275, and 1.332 MeV. Samples with the highest filler content (30 wt%) showed the best shielding ability. Considering the same filler content and energy level, the μ/ρ values of the nanocomposites are higher than those detected for the micro-composites. In particular, samples containing nano-Ta_2_O_5_ with lower filler content provided shielding performance comparable to that of high-loaded micro-composite systems. This can be justified by the high number of nanoparticles, with shorter interparticle distances than those of microparticles for the same volume fraction of fillers. This involves fewer void paths for photons from γ-rays, resulting in greater photon attenuation. The results unveiled that nano-Ta_2_O_5_ epoxy composites have higher thermal stability, flame retardance, and tensile properties compared to micro-composites at the same loading. Overall, these nanocomposites can be considered promising materials for effective γ-ray shielding in space.

A DGEBA epoxy resin was also used by Adeli et al. to fabricate neutron-shielding composites [52]. Boron carbide was used as filler, and the effect of the particle size (20 and 150 µm) and loading amount (1, 3, 5 wt%) on the shielding effectiveness was investigated. The results confirmed a shielding enhancement using smaller boron carbide particles. Other composites were prepared by adding tungsten oxide (WO_3_) and aluminum trihydrate (ATH) into the polymer matrix. The presence of WO_3_ provides protection against gamma rays, whereas ATH improves the heat resistance of the material. Moreover, ATH could also contribute to gamma-ray attenuation due to its higher atomic number than carbon and hydrogen in the epoxy matrix. Composites filled with WO_3_ and ATH showed an enhancement of more than 60% in neutron shielding compared to those filled only with boron carbide. Overall, these findings can be exploited for designing efficacious space shields.

Bel et al. tested composites based on poly(methyl methacrylate) (PMMA) reinforced with colemanite (CMT) as shielding materials for gamma rays and neutrons [53]. Samples with different CMT loadings (5, 15, 30, and 40 wt%) were irradiated using ^137^Cs and ^239^Pu-Be as gamma-ray and neutron sources, respectively. At higher filler contents, enhancements in the shielding effectiveness were found. Considering the gamma-ray irradiation tests, the addition of CMT at 40 wt% into the matrix led to an increase in terms of μ values from 0.098 to 0.138, which corresponds to 11.1% enhancement in shielding performance for the composite with respect to the neat PMMA. The results from neutron exposure experiments also confirm the best shielding effectiveness for the composites loaded with 40 wt% of CMT. This effect can be ascribed to the increase in terms of boron and hydrogen that are in the filler, thus allowing an increase in neutron attenuation. The PMMA/CMT composites were proposed as potential shields for satellites and space shuttles after further tests to be carried out on the International Space Station (ISS).

Composite materials based on poly-ether-ether-ketone (PEEK) and tungsten were fabricated for potential use as gamma-ray shields [54]. Samples were prepared by fused deposition modeling (FDM) 3D printing. The functional properties and shielding effectiveness of samples containing 50, 60, and 70 wt% of tungsten were investigated and compared with those of neat PEEK. ^60^Co and ^137^Cs were used as gamma-ray radiation sources. μ value of the neat PEEK exposed to ^137^Cs source was found to be 0.0728 cm^−1^. An increase in the μ value by 54.81%, 64.45%, and 68.70% was observed for samples filled with 50, 60, and 70 wt% of tungsten, respectively. After exposure to a high-energy ^60^Co source, the μ value of the samples reinforced with 50, 60, and 70 wt% of filler increased by 48.84%, 57.91%, and 62.91%, respectively, with respect to the pure polymer. The fabrication of multilayered hetero-structures based on PEEK/tungsten and PEEK/boron carbide was proposed for a contemporary shielding of neutrons and secondary gamma-ray radiations. The effective application of these composites in space could be evaluated, taking into account the advantages offered in terms of weight and costs. Polyether-polyurethane (PUR)-based composites filled with hexagonal boron nitride and amorphous boron were tested as neutron-shielding materials [55]. In particular, PUR composites loaded with 21% amorphous boron and boron nitride were fabricated. They showed suitable flexibility despite the high filler content and unveiled shielding effectiveness toward neutrons. These shielding properties and the possibility of using PUR matrix partly derived from renewable resources are factors that can be advantageously exploited in space applications.

Shemelya et al. fabricated 3D-printed polycarbonate (PC) composites filled with tungsten oxide for X-ray shielding [56]. Low loadings of filler were used to obtain a composite with suitable mechanical strength and reduced weight. After irradiation tests, an increase in X-ray attenuation (~10%) was observed for the composite with respect to pure PC. At low X-ray energies (40 keV to 70 keV), a tungsten volume loading of 0.3% results in an attenuation factor of 96 to 98 (rad/rad). The increase in terms of mass is ~5%, thus relatively low. Moreover, no significant changes in elongation to break or impact resistance were detected. Based on the results of this study, PC-based composites could be applied in space as shielding materials with suitable mechanical properties and low weight.

Table 1 summarizes the different PBM systems developed for space radiation shielding. The comparison is based on the type of polymer and filler that were used and on the shielded radiation.

## 3. Numerical Studies

The space environment poses significant hazards to both astronauts’ health and electronic devices, attributed to ionizing and non-ionizing radiation. Radiation transport codes are crucial in evaluating material shielding effectiveness before missions in the selection of appropriate radiation countermeasures. These codes are used for numerical simulations, offering insights into the interaction between charged particles and matter in space.

The radiation transport codes are broadly categorized into two types. First, deterministic codes are mainly used for one-dimensional (1D) problems but are adaptable to three-dimensional (3D) geometries, providing averaged dosimetric quantities. Then, Monte Carlo codes are exclusively employed for 3D investigations, and conducting statistical analysis when solving high-dimensional integral equations becomes challenging for deterministic methods [57]. The difference between deterministic and Monte Carlo approaches is significant. Deterministic codes are less demanding, but they can be used only if transport equations have analytical solutions. This is a disadvantage if the satellite has complex shielding geometries because of systematic errors in space discretization of the protective structure. On the other hand, Monte Carlo predictions are more difficult to implement because they require high processing power. Even if they cannot produce accurate results in radiation deep penetration problems, they are also able to simulate complex shielding geometries. Indeed, the main advantage of this method consists of creating numerical statistical solutions using “random” variables (of which the probability distribution is known) for a problem whose solution in closed form does not exist. Globally, deterministic and Monte Carlo methods complement each other and provide accurate results in space applications [58].

Different space agencies rely on codes developed by their national engineers that are tailored to their specific needs. Despite similar applications, these codes differ in aspects such as energy range and types of projectiles considered. A variety of software options can be found in the literature: the High charge (Z) and Energy TRaNsport (HZETRN) code and Online Tool for the Assessment of Radiation in Space (OLTARIS) software used by NASA, the SHIELD code by the Russian Space Agency, the GEometry ANd Tracking (GEANT4) code by ESA, the FLUctuating KAscade (FLUKA) code by the Italian Institute for Nuclear Physics (INFN) and the European Laboratory for Particle Physics (CERN), the Particle and Heavy Ion Transport code System (PHITS) code by the Japan Atomic Institute (JAERI), and the Monte Carlo N-Particle eXtended (MCNPX) code by Los Alamos National Laboratory [58,59,60].

### 3.1. Radiation Transport Codes

#### 3.1.1. HZETRN Codes

The HZETRN is a deterministic suite of Fortran codes pioneered at NASA Langley Research Center, which includes interactions and transports of ions from the galactic cosmic rays (GCRs), the trapped protons within the Earth’s magnetic field, and the energetic protons from solar particle events (SPEs). The codes work using high-charged particles to create different cross-sections as the first output for the selected materials and then transport energy inside the materials in free space and low earth orbit (LEO) [59,61,62]. The process is described by the 1D formulation of the Boltzmann transport equation obtained from conservation principles by considering a region of space filled with matter described by appropriate atomic and nuclear cross-sections [63]:

(9) $$\left[\Omega \cdot \nabla -\frac{1}{A_{j}}\frac{\delta }{\delta E}S_{j}\left(E\right)+\sigma_{j}\left(E\right)\right]\varphi_{j}\left(x,\Omega ,E\right)=\int \sum_{k}\sigma_{jk}\left(E,E^{\prime },\Omega ,\Omega^{\prime }\right)\varphi_{k}\left(x,\Omega^{\prime },E^{\prime }\right)d\Omega^{\prime }dE$$

where φ_j_(x, Ω, E) represents the flux of ions of type j with atomic mass A_j_ at position x moving in direction Ω. Sj(E) denotes the linear energy transfer (LET) as a function of energy E. σ_j_ and σ_jk_ (E, E′, Ω, Ω′) correspond to the reaction cross-sections for particle j with energy E and direction Ω, as well as the collision cross-sections between particles of type j with energy E and direction Ω, and type k with energy E′ and direction Ω′, respectively.

The code is structured into two sequential modules. In the initial “cross sections” module, input data related to the shielding material is processed to calculate atom fragmentation within the material. Subsequently, the “transport module” takes over and is responsible for guiding radiation energy through the shielding material. This module not only facilitates the transportation of radiation but also computes various dosimetric quantities based on the interactions between the material and the radiation that traverses it. By employing these modules in sequence, the code effectively models and analyzes the intricate behavior of radiation as it interacts with the selected shielding materials.

The HZETRN code has seen regular updates, with significant releases in 2010, 2015, and 2020, denoted as HZETRN 2010, HZETRN 2015, and HZETRN 2020, respectively. In HZETRN 2010, a 1D approach was employed using the straight-ahead approximation, which consists of transporting particles along a single ray (N = 1) representing the incident radiation. It also allowed for forward–backward (N = 2) propagation, where particles were propagated along a second ray at 180° relative to the incident beam direction [59]. HZETRN 2015 introduced 3D transport capabilities, allowing light ions (Z ≤ 2) to be propagated in three dimensions with an arbitrary number of rays (N) [59]. In contrast, HZETRN 2020 introduced a double solution, updating 1D and 3D transports. This version fully coupled muons and pions with neutrons and light ions and incorporated the Badhwaar–O’Neill (BON) 2020 model to accurately depict solar activity. Notably, the BON2020 model showed significant improvement over BON2014, eliminating systematic biases associated with the earlier version. The average relative error of the BON2020 model compared to available measurements was found to be lower than 1%. For heavy ion fragmentation studies, HZETRN 2020 offered two distinct models. The NUClear FRaGmentation (NUCFRG) model evaluated fragmentation cross-section products from nucleus–nucleus collisions, while the Relativistic Abrasion-Ablation FRaGmentation (RAADFRG) model predicted pre-fragment cross-sections [59,64,65].

#### 3.1.2. OLTARIS Software

The OLTARIS is a web-based space radiation analysis tool developed by NASA that employs the HZETRN code for radiation transport calculations and relies on the NUCFRG model for input data [60]. The software allows the investigation of the materials’ behavior considering different environmental conditions. Similar to the HZETRN code, OLTARIS simulates free space conditions, investigating the effects caused by GCR and SPEs at 1 AU. GCR is investigated using the BON model based on fitting the measured energy spectra. Furthermore, OLTARIS explores the behavior of materials on Martian and lunar soils through a 3D transport simulation. It enables the investigation of the combined effects of GCR and regolith (RG) on both Mars and the Moon. Specifically for Mars, the simulation also incorporates the combined influence of the atmosphere and RG in the boundary conditions. Furthermore, the software allows for the observation of radiation effects in LEO by considering circular orbits or customized trajectories. In this case, OLTARIS considers that the impact of galactic cosmic radiation (GCR) is influenced by orbit parameters, including inclination and altitude. Additionally, it accounts for the effects of trapped protons and neutrons reflected by the Earth’s albedo.

The software is divided into two main components. First, the website interface facilitates project creation and allows users to define custom materials and thickness distributions. The website interface is composed of five elements: radiation environment for definition of boundary conditions, material properties for user-defined materials, geometry of slabs, spheres, or vehicle thickness distribution, transport, and output results in terms of dosimetric quantities. Then, in the execution environment, Fortran executables are compiled on a computational cluster [66]. All user data are passed to the web server using Extensible Markup Language (XML) files.

#### 3.1.3. SHIELD Codes

The SHIELD was one of the first transport codes used in modern simulations. It is a Monte Carlo code developed by the Russian State corporation (ROSCOSMOS) for space flights and cosmonautics programs. The SHIELD code is tuned for space shielding and environment applications and was used for the simulation of radiation effects in 55 long-term spacecraft missions. The SHIELD code enables the transport of various particles, including nucleons, pions, kaons, anti-nucleons, and muons, along with nuclei having arbitrary proton and mass numbers at energies up to 1 TeV [67]. For charged particles, the code operates from a lower limit of 1 MeV, while for neutrons, it functions from their thermal energy level [58,59,67].

SHIELD operates through a simulated hadron cascade, where subatomic particles are transported to analyze the resulting secondary particles. These particles are stored in specific arrays with their individual parameters. Once the hadron cascade simulation concludes, SHIELD performs the transfer of neutrons with energies below 14.5 MeV from the source array using the original neutron transport code, LOw Energy Neutron Transport (LOENT) [68].

#### 3.1.4. GEANT4 Codes

GEANT4 is an open-source suite of codes written in C++, which uses Monte Carlo methods. It simulates a variety of physical interactions for high-energy nucleons, pions, and nuclei [59]. The codes contain different packages from the Quark Gluon String (QGS) model used for nucleons, pions, and nuclei to the Precompound (P) model referred to as nuclear de-excitation and the Bertini cascade (BERT) used for interactions below an energy of 10 GeV. Heavy ion collisions are modeled from the quantum molecular dynamics (QMDs) [69]. A novel package is PLANETOCOSMICS, which provides a description of several interesting features of a planetary body, including its geometric shape, the soil, the atmosphere, and the magnetosphere. It is mainly used for Mars, which is the current target of space exploration missions [58,70].

#### 3.1.5. FLUKA Code

FLUKA [71,72] is a Monte Carlo transport code written in Fortran by CERN and INFN [59], which is behind all beam-accelerator calculations [73]. It investigates the interaction and transport of hadrons, light and heavy ions, and trapped particles in electromagnetic fields in arbitrary materials (even with complex geometries). It is also used both for medical treatments and GCR and SPE environments [61]. Particle flux of protons and ion beams from 50 MeV/c to 450 GeV/c is simulated according to experimental data of the CERN accelerator [74].

#### 3.1.6. PHITS Code

PHITS [75] is a Monte Carlo suite of codes developed by JAERI and subdivided into two categories: transport and collision process. In the transport process, PHITS can simulate a motion under external fields, such as magnetic fields and gravity. One hypothesis is that without the external fields, neutral particles move along a straight trajectory with constant energy up to the next collision point. However, charge particles and heavy ions interact many times, with electrons in the material losing energy and changing direction. A secondary assumption is that the ionization process is not treated as a collision but as a transport process under an external field. The collision process occurs with the nucleus in the material and considers the decay of the particle.

#### 3.1.7. MCPNX Code

MCPNX is a Monte Carlo transport code distributed by the Radiation Safety Information Computational Center (RSICC) for the simulation of light-ion and particle interactions [60]. MCNPX has followed different updates denoted as MCNP5 and MCNP6. During energy transport, MCNP5 behaves like a stable code tracking neutrons, photons, and electrons. The output results are evaluated through nuclear data libraries, taking account of low-energy interaction probabilities [76]. The last version, called MCNP6, is clearly described by Rising et al. [77]. The code considers new neutron-induced fission systems and isotopes like ^240,242^Pu and ^252^Cf. In this way, MCNP6 can simulate secondary neutrons and γ-rays starting from a selected isotope through the default LLNL Fission Library [78].

#### 3.1.8. UPROP Code

The UPROP code is a deterministic transport code designed to model the nuclear fragmentation process using the straight-ahead approximation [61]. This approach involves numerically solving the one-dimensional propagation equation, assuming that all fragments maintain the same direction and energy per nucleon as the projectile nucleus. Notably, the code excludes considerations for the production or propagation of neutrons, leptons, or mesons.

### 3.2. Comparison of Strengths and Weaknesses of Radiation Transport Codes

The aforementioned codes are employed in radiation transport simulations, each with a distinct modeling approach. The effectiveness of each code varies, with strengths and limitations related to modeling capabilities and simulation speed. HZETRN may exhibit limitations in handling specific particle types or energy ranges, lacking detailed modeling of all secondary particle processes [79]. Similar issues are observed in OLTARIS, a web-based tool utilizing HZETRN codes. Both codes employ deterministic methods to simulate the transport of heavy ions and other particles in materials pertinent to space exploration [60,63]. GEANT4 models employ Monte Carlo methods to simulate the stochastic interactions of particles with materials, offering a detailed and adaptable tool for particle transport that can be used with multiple geometries and boundary conditions [58]. FLUKA utilizes Monte Carlo techniques to simulate electromagnetic and hadronic interactions for application in high-energy radiation shielding [80]. MCNPX and SHIELD utilize Monte Carlo methods to investigate nuclear reactions [78]. PHITS exploits Monte Carlo methods to simulate particle transport, incorporating physics models of magnetic and hadronic interactions [58]. UPROP simulates the proton transport but with lower performance than the other codes [81].

Lin et al. simulated the shielding behavior of polyethylene and aluminum semi-infinite slab shells of thickness between 0 and 160 g/cm^2^ [61]. Dose and equivalent dose were computed to assess differences between deterministic (HZETRN and UPROP) and Monte Carlo (GEANT4 and FLUKA) codes in SPE and GCR environments. For each thickness value, both materials consistently exhibited similar dose and equivalent dose values when computed using HZETRN, GEANT4, and FLUKA. However, these quantities are lower when calculated with UPROP for thicknesses exceeding 40 g/cm^2^. This discrepancy is attributed to the particles’ spectra sharing similar trends but featuring different energy values for each code. Proton fluence in HZETRN and in the two Monte Carlo codes is closely aligned, while UPROP showed significantly lower values, particularly below 10 MeV. As UPROP does not account for neutron transport, it is less accurate in computing dosimetric quantities and has not been actively utilized. Moreover, the neutron spectra provided by FLUKA and GEANT4 disagreed with HZETRN at low energies, specifically below approximately 5 MeV. Thus, a primary distinction among radiation codes arises from the energy values used to model particles’ spectra.

Aghara et al. investigated the primary and secondary particles resulting from interactions with an aluminum slab, specifically with average shielding thicknesses of 10 and 20 g/cm^2^ [60]. They conducted a comparative analysis involving the deterministic code, OLTARIS, and two Monte Carlo codes (MCNPX and PHITS). The study incorporated four models of SPE spectra: 56 Webber, 72 LaRC, 89 Weibull, and 91 Carrington. The fluence spectra of particles modeled by MCNPX and PHITS demonstrated significant agreement with OLTARIS results, particularly for protons and neutrons across all SPE environments. However, at lower energies (below 100 MeV), OLTARIS exhibited a lower neutron spectrum compared to MCNPX and PHITS. This difference is attributed to variations in atomic cross-sections and transport algorithms among the compared codes. Nuclear physics models in deterministic codes (such as HZETRN and OLTARIS) are limited, as they do not incorporate light-fragment particles in their cross-section models [59]. In contrast, Monte Carlo codes like SHIELD consider all generations of secondary particles, offering a comprehensive description of nuclear reactions across the entire energy spectrum of primary hadrons and nuclei up to 1 TeV/n. A comparison of the strengths and weaknesses of the codes is reported in Table 2.

Overall, radiation transport codes enable us to assess the combined effect of the radiation sources that characterize the space environment. The possibility to simulate different conditions that could be really experimented with during space missions represents one of the main strengths of the numerical approach.

### 3.3. Shielding Simulations for Radiative Environments

#### 3.3.1. GCR Environment

The fundamental task of numerical simulations performed in the GCR environment is the use of hydrogen-rich materials in order to amplify the attenuation of radiation passing through the matter [84]. This evidence comes from the Bethe–Bloch theory of materials’ stopping power based on experimental tests [85]. It was demonstrated that liquid hydrogen provides high shielding performance at minimizing the secondary particles produced from interaction with high atomic number energy (HZE) ions of GCR particles thanks to its elevated charge-to-mass ratio [9].

Polyethylene (PE) is considered the best solution for having hydrogen in a solid state because it is a non-toxic and stable material, even if it has flammability and outgassing problems [9]. The results in terms of linear energy transfer (LET) and dose were provided by Guetersloh et al. [20], performing simulations with Monte Carlo codes on the full GCR spectrum using a 1D transport model. Bragg curves were computed using a semiempirical model called BBFRAG [86,87] based on the Bethe–Bloch equation for energy-loss calculations and validated with the results of the accelerator experiment. The model has a single free modulation parameter Φ that defines solar activity, determining the GCR energy spectrum at the selected distance from the Sun [88]. They used Φ corresponding to the 2002–2003 period after the solar maximum to simulate a spectrum with a large fraction of the flux at high energy closer to the solar minimum. It was observed that fragments produce a dose absorption at depths beyond the Bragg peak as heavy-charged fragments are generated, demonstrating the shielding capability of PE.

**Figure 8:**
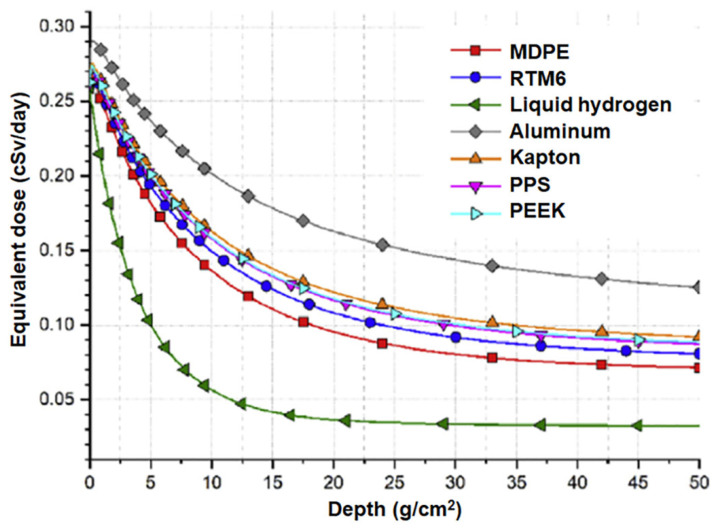
Equivalent dose for different materials as a function of thickness. Adapted with permission from Ref. [89].

Laurenzi et al. [89] investigated medium-density polyethylene (MDPE) using HZETRN 2015. A model of deep space (1 AU from Earth) based upon a 1D formulation of the Boltzmann equation for transport with a modulation parameter of 450 MV at solar minimum activity was adopted. They proposed a comparison between equivalent doses for MDPE, aluminum (Al), liquid hydrogen, and other polymers like Kapton, polyphenylene sulfide (PPS), polyether ether ketone (PEEK), and RTM6 epoxy resin. The materials were modeled considering the constituent atomic species, calculating the number of each atom per gram of material and the respective density. The results of numerical simulations are reported in Figure 8. A decreasing trend of the equivalent dose can be observed as the depth of the material increases. However, the shielding capability is far from the ideal behavior of liquid hydrogen. Al behaves worse than other materials, reaching a necessary mass that is two times higher than that of MDPE.

Similar results were provided for high-density polyethylene (HDPE) investigated by Barthel et al. [90] using HZETRN 2010. A comparison of slab and sphere models was obtained both for Al and HDPE, considering solar minimum conditions for a 500-day Mars mission. They found that 40–80 g/cm^2^ HDPE shows suitable shielding effectiveness against HZE ions simulating a human-manned mission if used as slab shells. In fact, the neutron flux generated from the interaction between GCR particles and Al (the main constituent material of a spacecraft) appears attenuated from the HDPE shield, leading to a dose absorbed by human tissue.

To enhance the mechanical properties of polyethylene, different researchers have tried to incorporate lightweight fillers like boron nitride nanotubes and carbon nanotubes with the aim of employing PE-based composites as structural and shielding materials. Specifically, these materials can integrate multifunctional mechanical, electrical, and thermal properties while demonstrating robust radiation-shielding capabilities in the space environment [91]. Relating to this objective, carbon and boron nanoparticles are incorporated in multifunctional materials, reaching superior modulus and optimal electrical and thermal conductivities [92]. Laurenzi et al. [89] have simulated composites made up of MDPE matrix and nanoparticles such as single-walled carbon nanotubes (SWCNTs), graphene oxide (GO), and boron carbide (CB_4_) reaching filler weight percentages of 1%, 2%, 5%, 10%, 15%, and 20%. They performed numerical calculations of the absorbed equivalent dose to investigate radiation attenuation against Al using HZETRN 2015. The results showed that the effects due to all fillers at low weight concentrations are negligible. It was also demonstrated that B_4_C and BN nano-powders provide suitable neutron shielding to PE composites. Kim et al. [12] investigated neutron attenuation through Monte Carlo simulations to evaluate the dependence of thermal neutron absorption on filler size for a boron-containing polymer composite. They observed that the neutron transmission is significantly less for an atomic cross-section of 300 μm, confirming the shielding efficiency of boron compounds.

Recently, micro- and nanocomposites made up of cis-polyacetylene matrix were considered in radiation shielding because they are optimal nanostructured hydrogen-storage materials [93]. Li-decorated polyacetylene and B-doped cis-polyacetylene can store up to 10.8 wt% and 9.8 wt% hydrogen, respectively [94]. For this reason, Yang et al. [95] have performed simulations to assess their protective effectiveness using MULASSIS (GEANT4 codes). A model simulating GCR radiation in deep space was used. They performed a 1D analysis considering a multilayered shield (thickness ranges between 2 and 50 cm). A comparison of the absorbed equivalent dose and fluences after shields in Al, liquid hydrogen, and doped polyacetylene with titanium (Ti), lithium (Li), and boron (B) was provided. It was denoted that Ti-doped polyacetylene plus 14% hydrogen was the most effective shielding material. Increasing the hydrogen percentage contained in Ti-doped polyacetylene, the production of neutrons decreases, reaching comparable particles’ flux to that of liquid hydrogen. Furthermore, the maximum flux in PE was over 0.04 particle/cm^2^, while the peak of Ti-doped polyacetylene was less than 0.03 particle/cm^2^. Regarding the comparison of equivalent doses, it was observed that polyacetylene composites have intermediate behavior between liquid hydrogen and Al with the same trend of PE.

**Figure 9:**
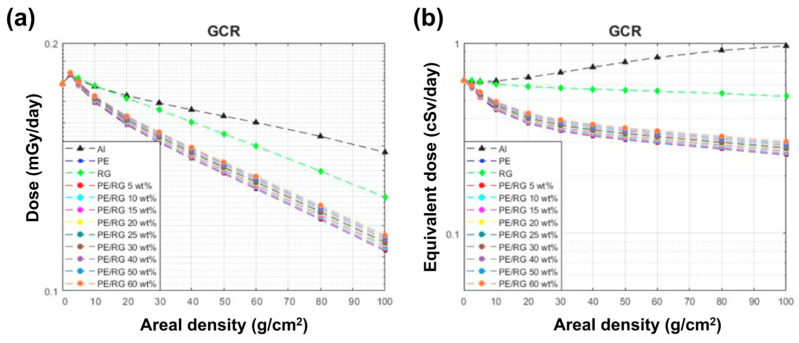
Comparison of dose and equivalent dose for Al, RG, PE, and PE/RG composites in different radiation fields at Mars ground level: (**a**) dose in the GCR spectra; (**b**) equivalent dose in the GCR spectra. Adapted with permission from Ref. [100].

GCR radiation also includes considerable ionizing particles for planets with negligible atmosphere and natural satellites. It is demonstrated that materials with low hydrogen content are more sensitive to radiation on Mars and the Moon’s surface when compared to polyethylene [96,97]. The inelastic collisions of neutrons and GCR ions with target atoms in pour hydrogen materials as Al increases the neutrons flux as the shield thicknesses rise. According to the literature, the in situ resource utilization (ISRU) strategy could reduce neutron damage by fabricating composite materials with Martian and lunar RG [98,99]. Zaccardi et al. [100] performed simulations about the shielding power of ultrahigh-molecular-weight PE (UHMWPE) composites. Simulations were conducted on sphere geometries at different altitudes and for different shielding materials. Radiation quantities at the target point (center of the sphere) were evaluated through a ray-by-ray transport procedure with HZETRN. They also demonstrated the improvement in mechanical properties by incorporating in situ Martian regolith in composite materials. Izod and flexural tests were carried out. The results of experimental analyses have validated the possibility of manufacturing structures with PE/RG composites. A comparison between Al, PE, RG, and PE/RG materials using OLTARIS software is reported in Figure 9.

All examined materials show a decrease in the absorbed dose with increasing the shield thickness. However, a different behavior was denoted for the equivalent dose, showing the shielding ineffectiveness of Al in the Martian environment. In fact, the equivalent dose increases with the areal density of the Al shield. This behavior determines a shielding gap between the deep space environment and the Martian surface. For this reason, PE/RG composites are considered protective materials since the absorbed equivalent dose decreases as the shield thickness increases. This result was confirmed by the neutron analysis. It was observed that neutron flux increases behind the Al shield while it remains constant considering the RG shield and decreases with PE and PE/RG 50 wt% shields. Al Zaman et al. [101] proposed innovative composites with Martian RG, whose shielding effectiveness was investigated through GEANT4 and OLTARIS. RG composite materials with poly-para-phenylene terephthalate (Kevlar), polyethylene terephthalate (Mylar), lithium hydride (LiH), polystyrene, and polypropylene were investigated. A preliminary analysis with GEANT4 was run to estimate the efficiency of radiation shielding in homogeneous and composite materials. The model consists of a 10 g/cm^2^ thick slab of RG placed outside, followed by a slab of selected materials (one by one) of variable thicknesses (1–5 g/cm^2^), a vacuum region, and a 5 g/cm^2^ of tissue region. Then, a spherical model was exploited in OLTARIS to calculate the effective equivalent dose for a female adult voxel (FAX) phantom (with “never smoker population” weighted tissue). Similar results regarding dose reduction have been noted in both the slab and sphere geometries. It is noteworthy that LiH/RG composites provide the highest dose reduction of about 9% with respect to the 15 g/cm^2^ Al shield. The other materials determine dose reductions between 5% and 9% when compared to the 15 g/cm^2^ Al shield. In this work, the use of 3D-printed RG composites was proposed not only for radiation protection but also for withstanding Martian storms and other harsh conditions on the planet.

#### 3.3.2. SPE Environment

**Figure 10:**
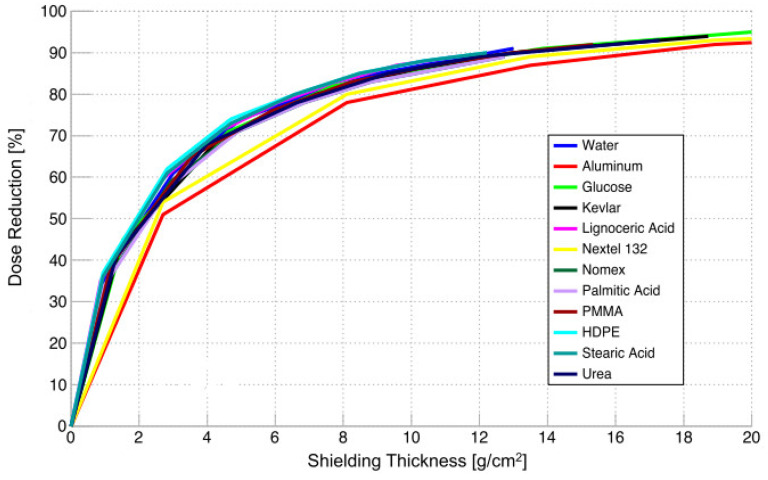
Percentage dose reduction in shielding materials as a function of thickness. Adapted with permission from Ref. [104].

SPEs are unpredictable and sporadic radiation coming from the Sun that occur near solar cycle maxima [102]. Liquid hydrogen is not only the best material to use against GCR, but it can also be employed as a protective material for protons and electrons, the main constituent particles of SPE spectra [103]. Different materials were simulated to assess their shielding ability. Vuolo et al. [104] have provided numerical analysis of materials for a wearable radiation protection spacesuit to mitigate the occurrence of acute radiation effects on sensitive organs. Simulations were performed with the GRASv3.3 tool (GEANT4), considering 1D and 3D setups. The SPE spectrum was calculated using the Emission of Solar Protons (ESPs) model of the ESA SPENVIS software (2015) for a 1-year mission without the Earth’s magnetic field to reproduce the deep space exposure [105]. Preliminary results have been computed for different materials selected on the base of their material index (MI) in 1D simulations. MI is defined as the ratio of the electronic stopping power to the nuclear interaction transmission. Figure 10 reports the percentage reduction in dose as a function of areal densities in g/cm^2^.

It is noteworthy that Al and Nextel 312, usually employed in space structures, offer the lowest dose reduction. At equal areal density, water, fatty acids, and PE show better shielding behavior. The highest MI was assigned to PE both from experimental and numerical results thanks to its high hydrogen content, followed by fatty acids and then water. Organic compounds were classified after water, followed by materials such as Kevlar, Nomex, and Mylar. Al and Nextel 312 have lower MI due to the high atomic number of their constituent elements. Three-dimensional simulations were performed to compare different models of spacesuits. Geometries were built using Geometry Description Markup Language (GDML) both for phantom and suit models. They considered a first model composed of water (ideal case) and a second multilayer made up of a containing layer of HDPE, Al or Kevlar, and an inner layer of water. Both models show a dose reduction of about 80% in extravehicular activity (EVA) and 44–57% in intra-vehicular activity (IVA) suits, reducing damage to blood-forming organs (BFOs).

PE and water are considered substantial candidates in both experimental and numerical studies. Laurenzi et al. [89] have investigated the shielding properties of MDPE and its compounds not only against GCR but also in the SPE environment using 1D simulations conducted with HZETRN2015. In particular, the difference between MDPE, PEEK, PPS, and Kapton appears minimal when comparing the absorbed equivalent dose after the Carrington event. Models of shielding materials were slab shells with thicknesses between 0 and 50 g/cm^2^. The discrepancy in shielding performance of polymers and Al is also reduced at high equivalent doses. However, it was demonstrated that reducing the damage associated with a single event requires a higher thickness of materials with respect to GCR. The shielding properties of PE nanocomposites with CB_4_, GO, and SWCNTs were also explored by evaluating the equivalent dose deviation from pure MDPE. The curves are constant at low filling loads as the thickness increases. Above 2 wt% of filler concentrations, the increase in the equivalent dose is reduced as a function of depth, demonstrating that fillers are less deleterious when larger thicknesses are considered. These results are particularly visible for composites with SWCNTs and GO. Iguchi et al. [106] developed a novel composite material consisting of benzoxazine resin with ultrahigh-molecular-weight PE (UHMWPE) fibers in order to reach a suitable compromise between shielding and mechanical performance. Materials were modeled employing the slab geometry option. Multiple simulations were conducted with slabs exhibiting an increasing areal density from 1 to 30 g/cm^2^. Equivalent dose of UHMWPE fiber/poly(3BOP-daC12) composite, Al, UHMWPE, Cycom 934 resin was calculated using OLTARIS for SPE free-space simulations of the August 1972 (King) event. It is evident that the hydrogen-rich poly-benzoxazine is approaching that of UHMWPE. It provides superior radiation-shielding properties compared to the Cycom 934 epoxy that is shifted even closer to the UHMWPE dose. To date, these UHMWPE/poly(3BOP-daC12) composites have been tested on the ISS as part of Materials International Space Station Experiment 12 (MISSE-12) from November 2019 to March 2020. The samples were installed externally to assess their behavior over a one-year period in space and to verify optical and tensile property degradation [107].

The shielding efficiency of water was primarily investigated in multilayered geometries. Aghara et al. [60] studied the radiation behavior of SPE spectra transporting through 10 or 20 g/cm^2^ Al shield followed by 30 g/cm^2^ of water slab. The results were validated using both deterministic (OLTARIS and HZETRN 2010) and Monte Carlo (MCNPX 2.7.0, PHITS 2.64) transport codes. The 56 Webber, 72 LaRC, 89 Weibull, and 91 Carrington SPE spectra were considered. The total particle fluence is similar for all SPE environments. Proton fluence decreases as thickness increases. On the contrary, photon fluence increases at high depths, leading to an increase in secondary photons produced from neutrons. The total equivalent dose was computed, including the contribution of neutrons. They observed that the contributions due to neutrons and photons are pronounced at higher thickness. The 91 Carrington event was more dangerous than the others, producing the highest amount of absorbed radiation, followed by 56 Webber, 72 LaRC, and 89 Weibull.

To date, metals are usually employed in space for shielding SPE spectra, even if they have low stopping power. Metal-based materials enriched with hydrogen and/or boron have been developed to reduce the thicknesses and volumes occupied by metals and achieve suitable shielding properties Rojdev et al. [108]. They investigated different materials using HZETRN 2010: metal hybrids (MHs), metal–organic frameworks (MOFs), and nano-porous carbon composites (CNTs) were loaded with hydrogen or methane for radiation protection considering Carrington event SPE spectra. The spectra were fitted using the band fitting method, and the differential spectrum was utilized as the input environment. Doses in tissue (cGy) were calculated for material thicknesses ranging from 1 to 100 g/cm^2^. All the materials perform better than Al, and only the hydrogen-loaded compounds outperform HDPE. However, they are all relatively close to the performance of HDPE.

Loffredo et al. [109] presented a study of protective materials made of Nomex doped with boron at 10, 20, and 30 wt% using GEANT4 for numerical simulations. They developed a numerical system setup by modeling experiments conducted at the NASA Space Radiation Laboratory. These experiments are based on the bombardment of an Al slab by a 1 GeV proton beam with the aim of reducing the dose from low-energy neutrons produced during the interaction of a 1 GeV proton beam with pristine Nomex [110,111]. As protons pass through the matter, there is a dose increase of 28% in the pure Nomex. This increase was reduced with the addition of boron, corresponding to a dose drop of about 14% in the best case (addition of boron at 10 wt%).

SPEs are dangerous for astronauts’ health also on Mars and the Moon’s surface even if their effects on human tissues are less strong than GCR. Exposures on the lunar surface are halved by the Moon itself, and on the Mars surface, they are reduced by over half because of the planet and the Martian atmosphere [112,113]. Zaccardi et al. [100] have examined absorbed dose on Mars’s surface using OLTARIS. The space environment was simulated considering the NASA LaRC model of August 1972 for SPEs. A comparison of Al, PE, and PE/RG composites was provided. As in the case of GCR, the absorbed dose demonstrates a decreasing trend as a function of increasing thickness. However, analysis of the equivalent dose shows the shielding ineffectiveness of Al. In fact, Al is effective only for small thicknesses due to the presence of neutrons. In contrast, RG has slightly better behavior than Al as the equivalent dose decreases. Overall, the effectiveness of PE/RG composites is demonstrated by dosimetric results in protecting against Martian radiation.

#### 3.3.3. LEO Environment

Radiation in LEO should be controlled by materials to implement passive shielding. In fact, this environment is affected by different sources of radiation. GCR and SPE radiation fields and trapped energetic electrons and protons (ERBs) are always present at these orbit altitudes [114]. The GCR and SPE fluxes are generally mitigated by the geomagnetic field, but they also depend on the solar activity [114,115]. The intensity of SPE spectra varies with the sunspot cycle on the solar surface. When solar flares and coronal mass ejections increase their periodicity and the radiation intensity emitted, the ionizing particles coming from the Sun can penetrate the Earth’s magnetic field and increase human illnesses caused by radiation exposure. The particle collisions with materials of space vehicles are dangerous due to secondary particles (neutrons) generated and then reflected by Earth’s albedo [116].

Laurenzi et al. [89] investigated the behavior of PE-based nanocomposite materials in LEO using HZETRN 2015. AP8 MAX trapped proton model for an orbit similar to the ISS orbit (inclination of 51.6° and altitude of 400 km) was used in numerical simulations. From a preliminary analysis, it was observed that polymers like PEEK, PPS, and Kapton have similar values of equivalent dose to each other and are better than Al shield. On the other hand, medium-density polyethylene (MDPE) shows the highest attenuation among solid materials, and therefore, MDPE-based nanocomposites were selected for the simulations. As in the case of SPEs, the equivalent dose of SWCNTs, GO, and CB_4_ composites decreases with increasing thickness, demonstrating better protection.

As for other space environments, the use of polyethylene is also relevant in LEO. Shavers et al. [117] developed a project to integrate PE into the International Space Station (ISS) crew quarters (CQs). This design is based on the “as low as reasonably achievable” (ALARA) requirement so as to ensure the safety of astronauts in space. A ray-tracing algorithm was developed to generate a shielding distribution for 812 directions geometrically placed around the center of each CQ. GCR and trapped particle fluxes were simulated with HZETRN for the ISS at 400 km altitude and 51.6° inclination. ISS materials were represented as Al. The results were reported only for the best case of PE shield with a thickness of 4.8 g/cm^2^. Simulations performed with HZETRN indicate that a 20% or more reduction in the equivalent dose is achievable.

Emmanuel et al. [118] have realized a layered structure made up of PE matrix and graphite (G) reinforcing fibers. This shield model was composed of alternating PE and graphite with 2, 4, 8, and 16 layers and a silicone detector to measure the total ionization dose (TID) using the GEANT4 codes. TID was simulated for a 15-year satellite mission in a highly elliptical orbit (HEO), specifically a Molniya orbit. GCR and SPEs were accurately modeled utilizing SPENVIS software, while trapped protons and electrons were defined by AP-8 MAX and AE-8 MAX models, respectively. One composite configuration, indicated as [PE/G]_2_ (with a 50% volume fraction of PE and an areal density of 1 g/cm^2^), has resulted in a TID lower than that of unfilled PE, demonstrating the possibility of tailoring the mechanical properties of PE-based composites with a minimal negative impact on its radiation-shielding effectiveness.

Recently, materials to be integrated into spacesuits for the individual protection of astronauts are being studied. Concerning EVA spacesuits, numerical simulations performed by Waller et al. [119] have been carried out with HZETRN, considering an orbit that follows that of the ISS for a duration of 10 years. The evaluated materials are summarized in Table 3. Input data comprise information on the density, number of atoms, atomic and mass number of each element. The results showed that only 9 cGy of equivalent dose is absorbed by astronauts during a mission to Mars while staying in parking LEO for a few days.

Atxaga et al. [11] fabricated graphite carbon composites with carbon nanotubes (CNTs) and tungsten nanoparticles as fillers and simulated their behavior for radiation shielding using GEANT4. Materials were defined using correction factors derived from experimental tests to estimate impurities in the layers. The simulations aimed to capture the overall dose absorbed from the electronic components of spacecraft in LEO. Specifically, the simulation setup involved slab geometries of shielding materials with different thicknesses (Al of 1 and 2 mm, tungsten of 50 and 100 µm, stainless steel of 50 µm and prepreg composite of 215 µm) followed by a silicon layer with a thickness of 300 µm. Promising results have been obtained for proton shielding (20 MeV). As predicted, the high-Z material, tungsten, had the most effect on the kinetic energy of the input protons. Layers made of prepreg have the same effect on particle energies as 50 µm tungsten. Overall, the prepreg layer is better or equal to that of 50 µm steel.

## 4. Summary and Challenges on the Design of Radiation-Shielding PBMs

### 4.1. Radiation Sources and PBM Design

High-energy photons from X-rays and gamma rays interact with matter, leading to absorption through energy transfer. These interactions are related to the atomic composition of the target materials and involve three main mechanisms: photoelectric effect, Compton scattering, and pair production. The photoelectric effect is most pronounced in high-Z materials at low energies (typically <500 keV) [92]. In the design of PBMs for radiation shielding, the use of nanofillers can be convenient to shield low-energy X-rays. In fact, several studies have demonstrated that nanostructured PBMs are able to absorb more low-energy photons compared to those reinforced with larger particle sizes [14,15]. This behavior can be ascribed to the increase in electron density within the material in the presence of nanoparticles. Similarly, for low-energy gamma rays, smaller, uniformly dispersed particle sizes offer greater photon blocking [16]. However, at higher energies, this effect decreases due to the transition from the photoelectric effect to Compton scattering. Lightweight and shielding-effective PBMs could be fabricated by incorporating suitable concentrations of high-Z fillers into a flexible matrix [92]. These fillers are able to attenuate radiation, whereas the use of a polymer matrix contributes to reducing the overall weight with respect to conventional shielding materials. Nevertheless, the use of high atomic number materials for shielding against GCR or SPE particles can lead to the emission of highly penetrating and detrimental gamma rays. Considering this, shielding PBMs should include elements that maximize projectile fragmentation while minimizing fragmentation of the target material. Polymer-based composites made of low-Z materials, particularly hydrogen or boron, have demonstrated efficacy in this regard [92]. Hydrogen, with its small atomic diameter, offers numerous interaction points in polymers for projectile fragmentation. Additionally, the absence of elements heavier than carbon minimizes the production of target fragments and, consequently, reduces the risk of secondary radiations. Overall, the aim is to strike a balance between effective shielding, reduced energy loss, and risks associated with secondary radiations during space missions.

Regarding neutrons, they interact mainly with atomic nuclei, penetrating deep into materials. In this case, interactions include scattering and trapping. Interactions with matter can lead to the generation of other radiations, such as protons, alpha particles, and gamma rays. In neutron shielding, hydrogen-rich polymers, such as PE, prove effective in attenuating fast neutrons. Boron-filled composites show remarkable thermal neutron-shielding efficiency. The balance between elements with light and heavy nuclei is crucial to fabricating effective neutron-shielding materials, although the difference in density between high and low-Z fillers poses challenges in achieving interfacial compatibility.

Structurally optimized designs with homogeneously dispersed fillers represent a challenge for the development of advanced radiation-shielding PBMs with multifunctional properties. The fabrication of these materials involves problems of agglomeration and non-uniform distribution of the functional reinforcement selected for shielding, compromising both efficiency in radiation protection and mechanical properties [120]. In this perspective, novel strategies to improve interface interactions can be adopted, such as opportunely mixing different fillers, surface modifications, and hybrid filler designs with shell structure. Nevertheless, considering the thermal scattering capacity, a multilayer structure could be adopted for realizing radiation-shielding PBMs, exploiting the ability of the different layers to efficiently scatter, reflect, absorb, and attenuate radiation.

### 4.2. Effects of Space Radiation on Polymers

Space radiation can potentially affect the properties of polymers, leading to alterations in their mechanical, thermal, and optical behavior [121]. Mechanical degradation can take place, with losses in flexibility and tensile strength. Thermal properties may be modified in terms of thermal conductance and energy stored in the material. Additionally, optical properties can be altered with changes in terms of emissivity and absorbance.

Typically, radiation interactions with polymers induce free-radical reactions [122]. Cross-linking and scission can take place with the generation of intermolecular bonds or backbone breaks, respectively. In particular, the presence of unsaturated bonds favors the occurrence of cross-linking after radiation exposure. Considering these effects, the addition of suitable fillers into the polymer matrix enables improved performances under ionizing radiation and effective shielding materials.

### 4.3. Other Constraints for the PBM Design

Strength, durability, and low weight are the additional requirements expected for space radiation-shielding materials. Moreover, these materials should maintain high performance until the end of the space mission. As explained by Shehab et al. [123], the total cost of the material includes recurring and nonrecurring fees. Recurring costs include raw materials, direct costs of working, and energy, while nonrecurring costs include indirect costs of working, equipment, tools, and facilities. In this context, PBMs can offer substantial advantages such as weight reduction, multifunctional properties, reduced number of parts needed to make the shielding component, and customizable strength and stiffness, which can lead to significant reductions in maintenance costs. PBMs can achieve a low “buy-to-fly” ratio, which indicates the amount of material used with respect to the final weight of the material after the fabrication process. Moreover, improved material testing is needed to better predict the long-term performances of these materials based on short-term testing [124]. In this perspective, advances in computational tools play a crucial role in predicting the response of materials to the combined effects of radiation in the harsh space environment.

**Figure 11:**
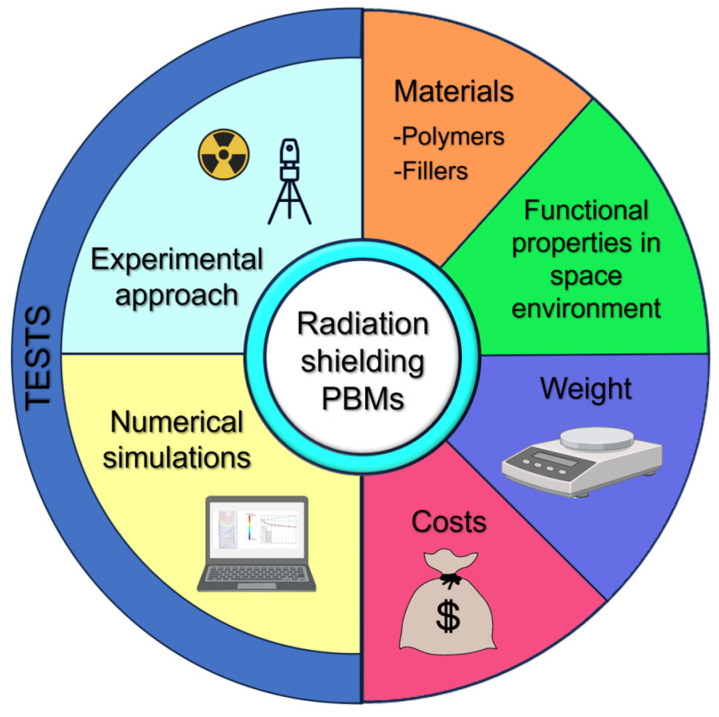
Graphical representation of the main issues related to the design of PBMs for space applications, reporting the two approaches for evaluating their shielding effectiveness.

Figure 11 summarizes the main issues related to the design of PBMs for space applications, reporting the two approaches for evaluating their shielding effectiveness.

## 5. Conclusions

Recent advances in the development of polymer-based materials (PBMs) with radiation-shielding properties were discussed, focusing on their potential application in the harsh space environment. The latest experimental studies on PBMs made of polyethylene (PE), polyimide (PI), polydimethylsiloxane (PDMS), and other functional matrices were described, considering the incorporation of different fillers. The effects of reinforcements such as boron nitride, boron carbide, carbon-based nanoparticles, and metals were considered. The optimization of the PBM design, as well as the suitable dispersion of the reinforcement, were taken into account for evaluating the overall performance of the PBMs. The role of the fillers on the radiation-shielding properties was widely discussed, and results compared with them were obtained for the neat polymer matrix under the same radiation exposure.

Numerical studies on PBMs, carried out by radiation transport codes, were argued. The codes were described as fundamental instruments for evaluating the material shielding effectiveness before space missions, as well as for the selection of the most suitable radiation countermeasures. Numerical analyses on PBMs in different space environments, such as GCR, SPE, and LEO, were examined. The results confirm the crucial role of the codes in predicting the shielding behavior of materials under different radiation conditions.

The relation between the different radiation sources and the development of efficacious shielding PBMs was discussed. Moreover, the main constraints on the design of suitable shielding materials were highlighted. New challenges are the optimization of the filler integration processes in the polymer matrix, the use of different fillers, and balancing the shielding effectiveness of the reinforcements and the multifunctional properties, which should be durable throughout the space mission. Furthermore, the development of novel advanced computational tools able to simulate the combined effect of the different radiation sources on spacecraft materials and biological tissues represents a further challenge for the definition of performing PBMs.

Overall, experimental studies are efficacious in providing detailed results on the effect of a specific radiation source on PBMs. Nevertheless, the combined effect of space radiation can be assessed using computational tools. The interaction between these two approaches is essential for the development of PBMs that can be successfully applied in the space environment.

In summary, this review provided an overview of the latest multifunctional PBMs that can be potentially applied in the harsh space environment as radiation-shielding materials. Crucial points in the definition of these materials and future challenges to improve them were also identified. By recognizing the evolving advances in this field, the objective of this review is to add value to the continuous endeavors aimed at defining the most effective PBMs for safeguarding both human health and spacecraft performance during space exploration.

## Figures and Tables

**Table 1:** Comparison of PBMs for space radiation shielding.

Polymer	Filler	Type of Shielded Radiation	Ref.
MDPE	Multiwalled carbon nanotubes, graphene nanoparticles	Protons	[22]
UHMWPE fibers, PU	Boron nitride	Neutrons	[23]
HDPE	Boron nitride, boron carbide	Neutrons	[25]
HDPE, LDPE	Hexagonal boron nitride	Neutrons	[27]
HDPE	Aluminum oxide, iron oxide, lead oxide	Gamma rays	[28]
PI	Bismuth oxide	Gamma rays	[33]
PI	Gadolinium oxide, hexagonal boron nitride	Gamma rays, neutrons	[34]
PI	Hexagonal boron nitride	Neutrons	[35]
PI	Lead	Electrons	[36]
FPI	Allomelanin nanoparticles	Ultraviolet	[37]
PDMS	Tungsten oxide, barium oxide	Gamma rays	[48]
PDMS	Bismuth oxide, multiwalled carbon nanotubes	Electrons	[49]
PDMS	Single-walled carbon nanotubes, detonation nanodiamond, zinc oxide	Protons	[50]
PDMS	Tungsten oxide, carbon nanotube sponge (sandwich configuration)	Gamma rays	[51]
DGEBA resin	Tantalum oxide	Gamma rays	[16]
DGEBA resin	Boron carbide, tungsten oxide, aluminum trihydrate	Neutrons	[52]
PMMA	Colemanite	Gamma rays, neutrons	[53]
PEEK	Tungsten	Gamma rays	[54]
PUR	Hexagonal boron nitride, amorphous boron	Neutrons	[55]
PC	Tungsten oxide	X-rays	[56]

Abbreviations MDPE: medium-density polyethylene; UHMWPE: ultrahigh-molecular-weight polyethylene; PU: polyurethane; HDPE: high-density polyethylene; LDPE: low-density polyethylene; PI: polyimide; FPI: fluorinated polyimide; PDMS: polydimethylsiloxane; DGEBA: diglycidyl ether of bisphenol A; PMMA: poly(methyl methacrylate); PEEK: poly-ether-ether-ketone; PUR: polyether-polyurethane; PC: polycarbonate.

**Table 2:** Strengths and weaknesses of radiation transport codes.

Radiation Transport Codes	Strengths	Weaknesses	Ref.
HZETRN OLTARIS	Accurate heavy ion transport simulation	Basic modeling of secondary radiation	[79]
GEANT4	Simulation considers stochastic interactions between particles and materials	Computationally intensive	[58]
FLUKA	Simulation of magnetic and hadronic interactions	Underestimation for proton irradiations below 10 MeV	[58,82]
SHIELD	Well-simulated nuclear reactions	Energy threshold level lower than 1 MeV for charged particles	[58,59]
PHITS	Simulation of magnetic and hadronic interactions	Ionization process is not treated as a collision but as a transport process	[58,75]
MCNPX	Well-simulated nuclear reactions	Time-consuming simulations	[68,83]
UPROP	Proton transport simulation	No neutron modeling	[81]

**Table 3:** Materials and their function inside the spacesuit.

Materials	Functions
Ortho-fabric	External cover
Aluminized Mylar	Insulator
Neoprene-coated nylon	Liner
Dacron	Restraint
Urethane-coated nylon	Pressure garment bladder (PGB)
Nylon	Liquid and cooling ventilation garment (LCVG)

## Data Availability

Not applicable.
